# Mapping TPACK self-efficacy beliefs in pre-service Chinese language teachers: a mixed-methods study

**DOI:** 10.3389/fpsyg.2026.1961246

**Published:** 2026-10-22

**Authors:** Baoqian Yang, Hongjuan Sun, Lifang Tang, Jia Cong, Ziqiao Wang, Shihan Wang

**Affiliations:** 1 Faculty of Education, Northeast Normal University, Changchun, China; 2 Tianjin Experimental Binhai Yuhua High School, Tianjin, China

**Keywords:** basic psychological needs, motivational functioning, pre-service teachers of Chinese as a foreign language, professional well-being, self-determination theory, self-perceived TPACK competence, technological pedagogical content knowledge

## Abstract

**Introduction:**

Digital education places distinctive psychological pressure on pre-service Teachers of Chinese as a Foreign Language (TCFL). This study reinterprets the seven-dimensional Technological Pedagogical Content Knowledge (TPACK) scale as a measure of self-perceived TPACK competence, read as an indicator of technology-integration self-efficacy beliefs.

**Methods:**

An explanatory sequential mixed-methods design was used. A total of 202 pre-service TCFL teachers completed questionnaires; eight were interviewed, and six lessons were video-coded (inter-coder κ = 0.83).

**Results:**

Self-perceived competence was unevenly distributed: Technological Knowledge was lowest (M = 3.68), and Technological Content Knowledge was highest (M = 3.83), the latter associated with short-term extrinsic online-teaching demands. Pedagogical Content Knowledge (*r* = 0.784) and Technological Pedagogical Knowledge (*r* = 0.835) showed the strongest associations with overall TPACK. Teaching practice, technology training, and competition experience each showed distinct associations, whereas gender and academic level did not. Interviews further surfaced two exploratory themes: employment pressure and cross-cultural online-teaching stress.

**Discussion:**

Self-Determination Theory is used only as an interpretive lens; basic psychological needs were not measured, so no claim about need satisfaction is tested. The findings point to pedagogical integration, rather than isolated technical training, as the priority for pre-service TCFL preparation.

## 1 Introduction

Pre-service teachers of Chinese as a Foreign Language (TCFL) face a distinctive burden. They must master logographic characters and intercultural instruction while also managing technostress, role ambiguity between language instructor and technical troubleshooter, and intense employment competition. Digital education has made online teaching proficiency a de facto entry requirement, so many trainees learn digital tools for career security rather than out of instructional interest (Ryan and Deci, 2020). These pressures bear directly on motivational functioning and on long-term professional well-being (Klassen and Chiu, 2010). This study starts from one central claim: scores on a TPACK self-report scale can be read as indicators of technology-integration self-efficacy beliefs. That reading matters. It turns a familiar knowledge taxonomy into a motivational-psychology instrument, so we can ask not only what trainees know, but how confident they feel about using technology in Chinese-language teaching and why uneven confidence carries motivational consequences.

The TPACK framework (Mishra and Koehler, 2006) is usually treated as a taxonomy of instructional knowledge. Most studies use its seven dimensions to catalog what teachers should know, not to indicate what teachers believe they can do. This matters for two reasons. First, the standard TPACK self-report scale (Schmidt et al., 2009) asks respondents to judge their own capability (“I can…,” “I know…”). Such items measure self-perceived competence, which is closely related to Bandurian self-efficacy and can be read as an indicator of technology-integration self-efficacy beliefs (Bandura, 1997). Second, that self-efficacy reading is what allows motivational questions to be asked at all. If TPACK scores index confidence rather than knowledge, then uneven scores index uneven motivational belief, and uneven belief has consequences for well-being. We develop this argument in Section 2.1 and return to it throughout. We note that perceived competence is not psychometrically identical to self-efficacy; our claim is interpretive, not a claim of measurement equivalence. Despite this potential, TCFL research has rarely used TPACK data to examine motivational mechanisms, and few mixed-method designs have unpacked the affective processes behind scale scores (Dong, 2023).

Four specific gaps follow from this motivational reading of TPACK. First, while substantial evidence links self-efficacy to teaching behavior in general education, the motivational belief structures of pre-service teachers in logographic language instruction remain unexplored, despite the unique cognitive and affective demands of Chinese character and tone pedagogy. Second, existing studies have documented correlations between TPACK dimensions and various contextual factors, but few have interpreted these relationships through the lens of basic psychological need satisfaction (Ryan and Deci, 2020)—that is, whether teaching practice, training, or competitions satisfy or frustrate the innate needs for autonomy, competence, and relatedness that underpin sustained intrinsic motivation. Third, the affective moderators that mediate or moderate the link between TPACK beliefs and professional well-being particularly career anxiety and cross-cultural teaching stress have been almost entirely neglected in quantitative TPACK surveys. Fourth, prior research rarely examines how imbalanced self-efficacy belief profiles might accelerate emotional depletion and undermine long-term career commitment among pre-service language educators.

Against the above research context and identified literature gaps, this study adopts explanatory sequential mixed methods grounded in Bandura’s social cognitive theory and Self-Determination Theory, taking the seven-dimensional TPACK scale as a standardized measuring instrument of technology integration self-efficacy. The research recruits two hundred and two pre-service TCFL as formal participants, with three core research objectives established. First, to comprehensively map the overall distribution of trainees’ TPACK based technology integration self-efficacy belief systems and summarize motivational deficits reflected by uneven dimensional scores. Second, to systematically sort the motivational-cognitive correlation structure across all seven TPACK sub-dimensions and examine whether PCK and TPK appear to function as psychological anchors supporting long-term intrinsic digital teaching motivation. Third, to examine associations between demographic / experiential contextual variables and self-perceived TPACK competence, and explore, via qualitative interviews and classroom observation data, potential perceived contextual barriers and exploratory qualitative affective themes that may shape motivational functioning and long-term professional well-being. Basic psychological need satisfaction was not directly measured in this study; situational factors are interpreted through Self-Determination Theory as a post-hoc theoretical framework rather than empirically tested predictors.

Three standardized psychological-oriented research questions guide the full analysis process:

1. What imbalances exist across the seven dimensions of self-perceived TPACK competence measured by the TPACK scale, and what intrinsic and extrinsic motivational interpretations may be offered for relatively high and low subscale scores?
2. What motivational-cognitive correlations exist among all TPACK dimensions, and to what extent do PCK and TPK beliefs appear to function as psychological anchors for sustained digital teaching engagement?
3. Which demographic and experiential factors are associated with pre-service teachers’ self-perceived TPACK competence, and what potential affective themes (identified from qualitative accounts) may relate to their motivational functioning and professional well-being?

This research theoretically reconstructs the psychological connotation of the TPACK framework and shifts analytical focus from instructional technology to motivational psychological processes; methodologically, it combines large-scale psychometric data with motivational thematic interviews and classroom behavioral coding to capture implicit affective mechanisms inaccessible to single questionnaire designs; contextually, it fills the research vacuum of motivational psychology for cross-cultural pre-service educators and delivers targeted psychological intervention strategies to regulate motivational states, consolidate adaptive pedagogical beliefs and mitigate occupational psychological exhaustion.

## 2 Literature review

### 2.1 Conceptual clarification: TPACK knowledge, self-perceived competence, and self-efficacy

Three constructs are often conflated in TPACK research, and distinguishing them is essential for everything that follows. Actual TPACK knowledge is what teachers objectively know about integrating technology, pedagogy and content; measuring it would require performance-based assessment. Self-perceived TPACK competence is a trainee’s subjective assessment of their own knowledge and capability; this is what the Schmidt et al. (2009) scale and our adapted instrument measure. Bandurian self-efficacy (Bandura, 1997) is a narrower, context-bound judgment of one’s capability to organize and execute the actions required for a designated performance. Our items use self-perceptual wording (“I can…,” “I know…”), which captures perceived competence. We therefore read TPACK scores as indicators of technology-integration self-efficacy beliefs. This is a theoretical interpretation, not a claim of psychometric equivalence. We place this distinction at the outset because every later interpretation in the paper depends on it.

### 2.2 Bandura’s social cognitive theory and teacher technology integration self-efficacy

Self-efficacy serves as the core explanatory variable of Bandura’s social cognitive theory, which defines the construct as individuals’ stable subjective judgment of their own capacity to complete domain-specific complex tasks (Bandura, 1997). Within educational contexts, technology-integration self-efficacy refers to educators’ inner confidence in coordinating digital tools, subject content and instructional strategies to deliver complete lessons. According to this theoretical framework, self-efficacy beliefs are shaped by four primary formative sources: mastery experience from authentic practice, vicarious experience from observing peer teaching, verbal persuasion from supervisors or peers, and emotional arousal generated during task execution. Negative emotional arousal, such as repeated technical failures during instructional activities, may suppress self-confidence and reduce willingness to engage in technology-enhanced teaching (Scherer et al., 2019).

Meta-analytic research rooted in social-cognitive theory has demonstrated that technology-integration-related self-efficacy exerts stronger predictive power over educators’ voluntary digital tool adoption than hardware resources or formal training duration (Scherer et al., 2019). It should be noted that these theoretical propositions are general psychological assumptions, and they require domain-specific empirical verification for different teacher populations. Most TPACK-oriented research draws on this self-efficacy framework, yet many studies treat TPACK dimensions purely as knowledge taxonomies rather than psychological belief indicators, creating space for further motivational-psychological analysis (Dong, 2023).

### 2.3 Self-determination theory and three basic psychological needs

Within Self-Determination Theory proposed by Ryan and Deci (2020), motivation is conceptualized along a continuous autonomy-oriented regulatory continuum. At the fully autonomous end lies intrinsic motivation, stemming from spontaneous interest in designing customized Chinese digital courses. Extrinsic motivation is not a unitary opposing category; instead, it comprises multiple forms of behavioral regulation varying in degrees of internalization, including external regulation, introjected regulation, identified regulation and integrated regulation. These different regulatory styles originate from external pressures such as employment assessment, graduation requirements and teaching competition awards, yet they can internalize to different extents.

The core argument of this theory holds that long-term sustainable intrinsic motivation can only be cultivated when three innate psychological needs are continuously satisfied: the need for autonomy (the right to independently design lesson plans and select digital resources), the need for competence (continuous positive feedback on teaching performance), and the need for relatedness (collaborative communication with peers and overseas learners). Traditional tool-centered technical training widely adopted by universities may primarily satisfy superficial competence-related experiences and offer limited opportunities for autonomous curriculum design; such contexts tend to favor less internalized extrinsic regulatory styles, though this outcome is not inevitable (Voogt et al., 2016). Prior TPACK literature has rarely adopted this three-needs analytical framework to interpret the differentiated motivational effects of teaching practice, training and competitions, which this research intends to address.

These theoretical premises provide a conceptual lens for interpreting group differences observed in empirical data, though they do not by themselves constitute domain-specific evidence for teacher populations.

### 2.4 Pedagogical beliefs and educators’ long-term professional well-being

Pedagogical beliefs refer to stable long-term cognitive judgments held by educators about subject content, instructional strategies and digital teaching resources. Imbalanced belief systems may produce persistent psychological strain and contribute to occupational-burnout-related risk (Klassen and Chiu, 2010). From a general teacher-education perspective, professional well-being comprises three inter-related psychological components: active instructional engagement intention, low risk of emotional exhaustion, and stable long-term vocational identity.

When educators hold fragmented or low-confidence pedagogical beliefs, they may experience frequent self-doubt in instructional practice, which in turn can elevate burnout risk and reduce long-term career willingness (Tondeur et al., 2017). Existing general-population teacher research has emphasized that curriculum reform and technical training alone cannot guarantee positive motivational outcomes; psychological states and belief structures also play a critical role. This general theoretical framework can be applied as an interpretive tool for pre-service-teacher research across disciplines.

### 2.5 Reinterpreting TPACK as a psychological measurement of self-efficacy beliefs

Mishra and Koehler first proposed the TPACK theoretical framework in 2006 to describe the professional knowledge system required by educators implementing technology-enhanced instruction (Mishra and Koehler, 2006). The classic three-circle Venn model defines seven inter-related dimensions situated within diverse contextual constraints, as illustrated in Figure 1.

**Figure 1:**
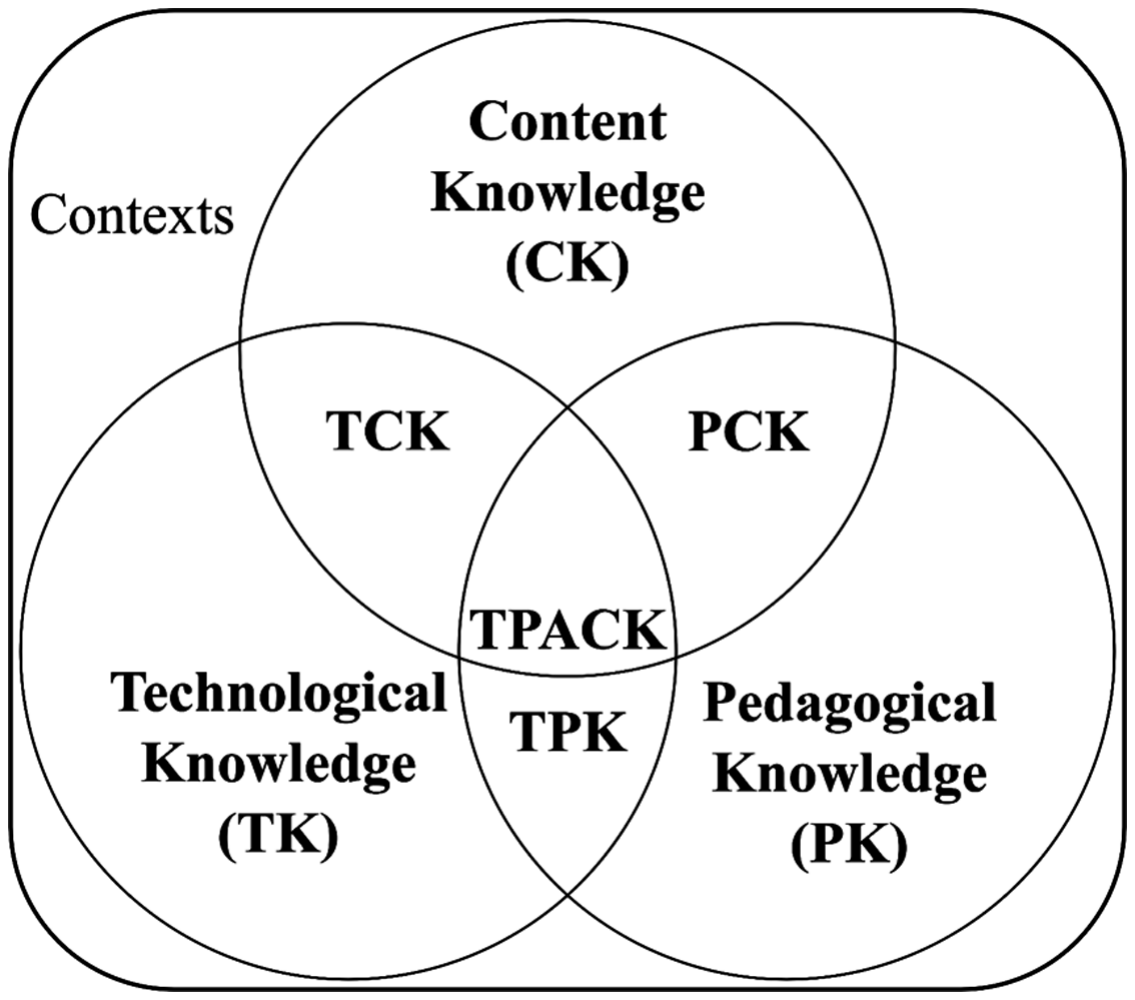
Seven-dimensional TPACK self-perceived competence structural framework (Mishra and Koehler, 2006).

On the basis of this structural model, Schmidt et al. (2009) developed a seven-dimension self-report questionnaire, which measures self-perceived TPACK competence—a construct that captures individuals’ subjective assessments of their own knowledge and capabilities across the seven TPACK domains. While self-perceived competence is closely related to Bandurian self-efficacy (Bandura, 1997), which refers to individuals’ judgments of their capabilities to organize and execute courses of action required to attain designated types of performances, the two constructs are not psychometrically identical. Self-efficacy is more specific, context-bound, and focused on the judgment of capability to execute specific tasks, whereas perceived competence is a broader self-assessment of knowledge and ability. Throughout this paper, we interpret scores on the TPACK self-report scale as indicators of technology-integration self-efficacy beliefs, recognizing that this interpretation is a theoretical framing rather than a claim of psychometric equivalence.

It is important to distinguish among three related but distinct constructs that are often conflated in TPACK research. First, actual TPACK knowledge refers to what teachers objectively know about integrating technology, pedagogy, and content, a construct that would require performance-based assessment to measure directly. Second, self-perceived TPACK competence refers to individuals’ subjective assessments of their own TPACK knowledge and capabilities; this is what the Schmidt et al. (2009) scale and our adapted instrument actually measure. Third, Bandurian self-efficacy (Bandura, 1997) refers to individuals’ judgments of their capabilities to organize and execute courses of action required to attain designated types of performances, a more specific, context-bound construct focused on the confidence to execute particular tasks in particular situations. The TPACK self-report scale items use self-perceptual language (“I can…,” “I know…”) that captures perceived competence, which is closely related to but not identical with self-efficacy. In this study, we interpret scores on the TPACK scale as indicators of technology-integration self-efficacy beliefs, recognizing this as a theoretical interpretation grounded in Bandura’s framework rather than a claim of psychometric equivalence. This interpretation is consistent with a growing body of research that treats TPACK self-report measures as proxies for self-efficacy beliefs (Scherer et al., 2019; Dong, 2023), but we acknowledge that direct measurement of self-efficacy would require more context-specific and task-specific items.

Four lines of evidence support this reading. First, item wording: the Schmidt et al. (2009) items ask respondents to judge their own capability (“I can…,” “I know…”). That is the judgment format Bandura (1997) prescribes for self-efficacy scales, rather than a request to demonstrate knowledge. Second, scale-development practice: validation work on TPACK self-report instruments has repeatedly framed them as measures of efficacy or confidence (Koh et al., 2013; Koehler et al., 2013), and reviews of the field routinely treat TPACK self-reports as self-efficacy measures (Scherer et al., 2019). Third, predictive function: TPACK self-reports predict teachers’ voluntary technology use better than objective indicators of competence or of resource access (Scherer et al., 2019; Schmid et al., 2021). That pattern matches how self-efficacy is theorized to operate, and not how knowledge is theorized to operate. Fourth, convergent interpretation: studies of pre-service teachers describe TPACK self-report profiles in terms of confidence and perceived challenge rather than in terms of tested knowledge (Valtonen et al., 2020; Dong, 2023). Taken together, these arguments justify reading TPACK self-report scores as indicators of technology-integration self-efficacy beliefs. They do not establish measurement equivalence. Perceived competence remains broader and less task-specific than Bandurian self-efficacy, and direct measurement would require more context-specific items.

Combined with subject-specific teaching characteristics, researchers can further define the connotation of each dimension for particular learner groups. Table 1 shows the dimension-specific connotations adapted for the research context of this paper.

**Table 1:** Specific connotations of seven TPACK self-perceived competence dimensions for pre-service TCFL.

Specific connotations	
TK	Knowledge of modern educational technology (e.g., PPT creation, audio-visual editing, usage of online teaching platforms).
CK	Mastering relevant knowledge in linguistics, intercultural communication, Chinese culture, pedagogy, and psychology.
PK	Mastery of theories of second language acquisition and knowledge of second language teaching methodologies (e.g., Task-Based Instruction, Communicative Approach, etc.).
PCK	Acquire knowledge of specific teaching methods and strategies for teaching Chinese as a second language.
TCK	Master the knowledge of applying information technology to the presentation of Chinese teaching content and assessment processes (e.g., utilizing pinyin annotation software, Chinese language teaching databases, etc.).
TPK	Grasp the knowledge of selecting, processing, and producing technological resources (e.g., courseware, animations) based on Chinese teaching requirements to optimize teaching methods.
TPACK	Mastering the knowledge of effectively integrating subject content, teaching methods and modern educational technologies in teaching Chinese as a foreign language, in order to create an efficient learning environment.

Table 1 systematically matches each TPACK dimension in Figure 1 with its corresponding self-efficacy psychological meaning in the Chinese language teaching context. TK self-efficacy reflects trainees’ confidence in operating and troubleshooting Chinese-specific digital tools, while CK self-efficacy represents stable identity beliefs of Chinese linguistics and cross-cultural knowledge. PCK and TPK are defined as two core motivational cognitive hubs that support intrinsic digital teaching motivation, which lays the theoretical foundation for subsequent correlation analysis of scale data (Luo, 2022). Most early TPACK literature only interprets each dimension from the perspective of teaching content division, ignoring the psychological measurement attribute of the scale, and few studies have formulated discipline-specific self-efficacy definition tables for Chinese language teaching as shown in Table 1, resulting in insufficient contextualization of TPACK motivational research targeting Chinese pre-service teachers (Zou et al., 2022). This conceptual reorientation from viewing TPACK as instructional knowledge taxonomy to interpreting it as a self-efficacy belief system carries substantive implications for motivational psychology research. When a pre-service teacher reports a low TK score, it is not merely a statement of limited technical proficiency; it is a psychological signal of diminished confidence that, when chronic, may activate avoidance motivation and erode teaching engagement. Similarly, high TCK scores driven by mandatory online teaching requirements reflect externally regulated motivational orientations that, according to SDT (Ryan and Deci, 2020), are inherently less stable and more vulnerable to psychological depletion than autonomously regulated motivation. This reconceptualization frames the subsequent empirical analyses not as assessments of teaching competence, but as investigations of motivational belief systems and their psychological antecedents and consequences.

### 2.6 Conceptual model: predictive variables, hidden negative perceived contextual barriers and exploratory affective themes

Drawing on Bandura’s social-cognitive theory, Self-Determination Theory and the TPACK framework described above, this section theoretically deduces potential relationships among key variables to construct the conceptual model presented in Figure 2. From our interpretive stance, existing literature mostly discusses demographic, experiential, and affective risk factors in isolation. Few studies have systematically synthesized these three categories of predictors within a unified motivational framework for pre-service TCFL teachers; questionnaire-only designs also cannot capture latent affective barriers, which provides strong rationale for the mixed-methods approach adopted in the present study. From a purely theoretical perspective, two broad sets of factors may shape educators’ self-perceived TPACK competence.

**Figure 2:**
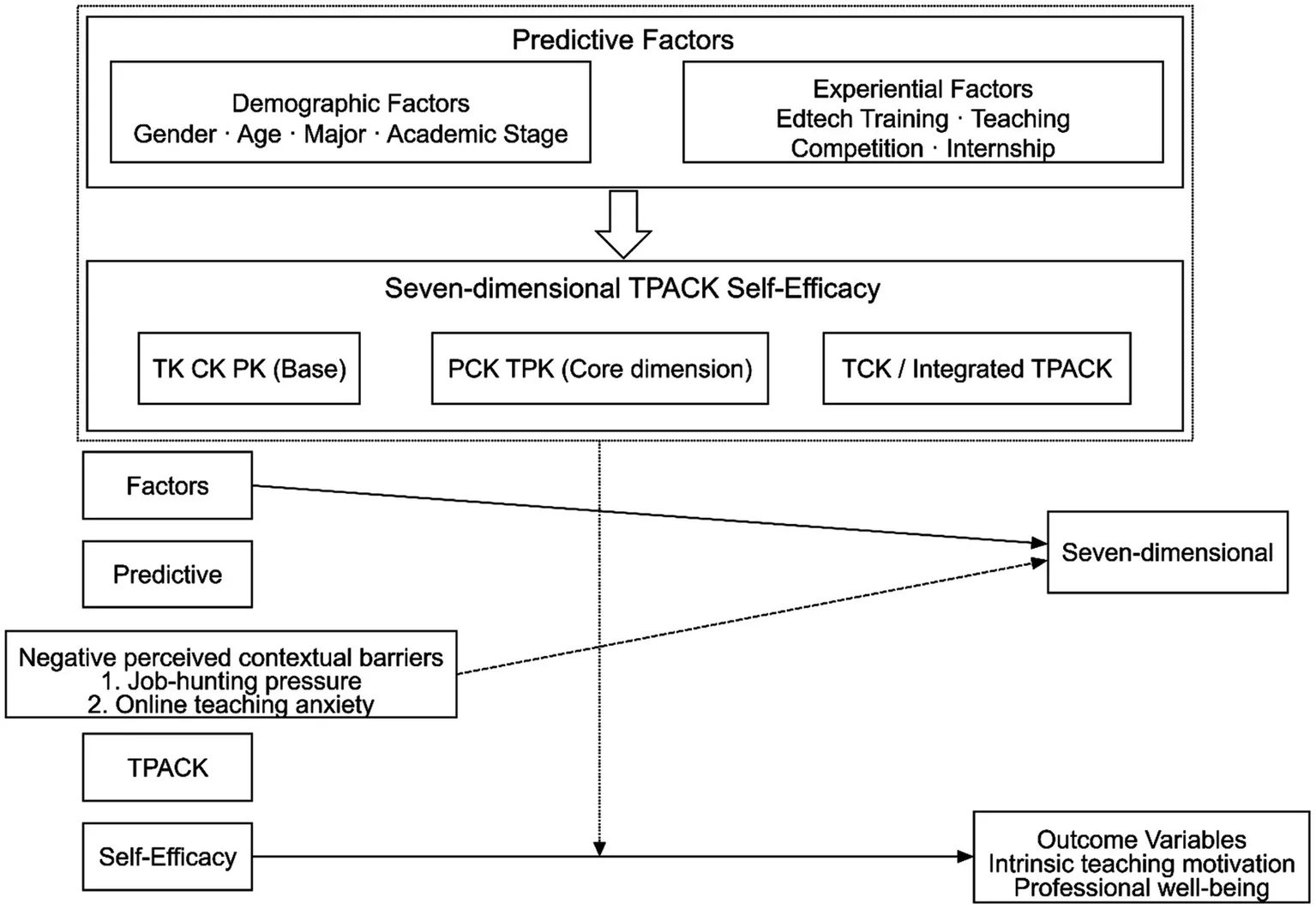
Integrated theoretical model of pre-service TCFL teachers’ TPACK motivational mechanism.

#### 2.6.1 Individual demographic psychological variables

Static demographic background variables (gender, age, academic stage, undergraduate major) may correlate with differences in belief systems, because different background conditions offer divergent learning and practice opportunities. In our reading of prior scholarship, although separate empirical studies have reported group differences linked to these demographic variables, inconsistent findings still exist across different teacher populations. Therefore, it remains necessary to test whether these theoretical expectations hold for the specific sample of pre-service TCFL teachers. Demographic variables are treated here as fixed classificatory factors: they describe who the trainees are, and they are not directly open to intervention.

#### 2.6.2 Environmental experiential motivational stimulating factors

Experiential variables differ from demographic variables in one decisive respect: they are malleable. Training, teaching-skill competitions and teaching-practice tenure are conditions that teacher-education programs can create, extend or redesign. They may therefore afford or frustrate autonomy-, competence- and relatedness-related experiences, and may be associated with variation in self-perceived TPACK-related beliefs. Because they can be deliberately arranged, experiential factors form the primary target of the practical recommendations developed in Section 5.5. Demographic factors, by contrast, mainly serve to describe sample composition and to check for confounding.

#### 2.6.3 Hidden negative affective psychological moderators

The model further theoretically posits a set of hidden negative perceived contextual barriers. These affective and contextual risk factors are not directly captured by standard TPACK questionnaires. Theoretically, such perceived barriers may produce emotional depletion and constitute risk factors for educators’ professional well-being.

Notably, most mainstream TPACK quantitative research tends to overlook these non-questionnaire-measurable affective barriers. If we rely merely on survey data, these important risk mechanisms would remain invisible, reinforcing the value of supplementary qualitative inquiry via interviews and classroom observation.

Figure 2 should be read as follows. The boxes on the left represent the two predictor families described above, demographic and experiential. The box at the center represents self-perceived TPACK competence across the seven dimensions. The boxes on the right represent the motivational, affective and well-being-related outcomes discussed in Section 2.4. Solid arrows denote theoretically hypothesized associations that the quantitative phase tests directly, running from predictors to self-perceived competence. Dashed arrows denote pathways that the quantitative data cannot test and that we examine only through qualitative material, running from self-perceived competence to motivational and well-being-related outcomes. Dotted arrows denote paths proposed in the literature but not examined in this study; they are drawn to make the boundaries of our design explicit. No arrow implies causation. Every path is a correlational expectation derived from the theories reviewed above.

It should be emphasized that the relationships described in this section represent theoretically-derived hypothetical pathways, rather than empirical findings from the present dataset. Subsequent quantitative and qualitative analyses will examine whether patterns observed in our sample are consistent with these theoretical expectations. The integrated theoretical predictive model is presented in Figure 2.

### 2.7 Empirical research on TPACK and motivational functioning among pre-service TCFL teachers

Growing empirical work has begun to examine TPACK-related belief structures among pre-service Teachers of Chinese as a Foreign Language, yet motivational-psychological perspectives remain comparatively under-utilized within this domain (Dong, 2023; Luo, 2022). Most existing TCFL-oriented TPACK investigations focus on curriculum design, training effect evaluation and descriptive knowledge status statistics, while few studies center pre-service teachers’ subjective self-perceived competence and associated motivational-affective experiences (Zou et al., 2022).

Existing TCFL-specific empirical findings indicate that pre-service Chinese-language educators exhibit imbalanced TPACK-related self-perceived profiles. Technical-knowledge-related self-perceived competence is frequently reported as a relative weakness, whereas content-technology matching (TCK-related) confidence is comparatively high, often associated with external task-driven online-teaching requirements (Liu and Wang, 2022; Dong, 2023). Within TCFL cohorts, pedagogical-content knowledge (PCK) and technology-pedagogy knowledge (TPK) consistently show strong correlations with overall integrated TPACK self-perceived competence, which has drawn scholarly attention to the central role of pedagogical belief systems for language-teaching contexts (Luo, 2022).

Demographic and experiential factors also display domain-specific patterns among TCFL pre-service teachers. Existing TCFL empirical studies report that teaching practice, educational-technology training and teaching-skill competitions are associated with differentiated TPACK related self-perceived competence. At the same time, pre-service TCFL participants commonly face unique stressors: employment-market-related anxiety and cross-cultural online-teaching barriers, including time-zone conflicts and unstable network environments (Dong, 2023; Zou et al., 2022). These context-specific affective barriers cannot be adequately captured by generic TPACK questionnaires and require supplementary qualitative investigation.

Nevertheless, the existing TCFL empirical literature still contains evident research gaps. Few mixed-method studies systematically connect TPACK self-perceived competence to motivational-functioning constructs derived from Self-Determination Theory. Direct quantitative measurement of basic-psychological-need satisfaction is rare in existing TCFL datasets. In addition, most published TCFL TPACK studies adopt cross-sectional designs and therefore cannot disentangle correlational associations from causal developmental processes; longitudinal research tracking motivational changes across pre-service training pathways remains scarce (Dong, 2023). Against these identified domain-specific empirical gaps, the present mixed-methods study is developed.

## 3 Research methodology

All questionnaire, interview and classroom-video data were collected in 2022. The 2022 field-work was reviewed and approved at faculty level by the Faculty of Education, Northeast Normal University, under the internal procedure then applicable to low-risk educational survey research. Under that procedure the study was exempt from full institutional review board review, because it involved anonymous or confidential educational survey and classroom-observation data, no sensitive personal information and no intervention. No certificate number was issued in 2022. For each data-collection component we obtained consent in writing: an online checkbox consent before questionnaire participation; signed consent forms before interview audio-recording; and signed consent for use of classroom-teaching video recordings. All questionnaire responses, interview recordings and teaching videos were fully anonymised. Because the journal requires a formal approval record, we subsequently obtained institutional ethical clearance (Approval No. 202402036) in 2024. That clearance covers secondary analysis and publication of the already-completed, anonymised 2022 dataset only. No new data were collected after the approval was granted.

This study adopts explanatory sequential mixed-methods design proposed by Creswell and Plano Clark (2017). The research proceeds in two ordered phases. Phase 1 (quantitative): Questionnaire data from 202 participants were first collected to map group-level associational patterns across seven TPACK self-perceived competence dimensions. Key Phase 1 statistical outputs guided Phase 2 qualitative work: purposive sampling of interviewees and classroom-observation participants was stratified according to meaningful quantitative subgroup patterns (e.g., high/low TCK scores, with/without training/practice experience). Furthermore, the semi-structured interview outline and thematic-analysis analytical priorities were adjusted to focus on interpreting those statistically meaningful quantitative associations. Phase 2 (qualitative): From the questionnaire respondents, eight participants were selected for semi-structured interviews, and six participants were selected for classroom video observation. Among them, four individuals participated in both semi-structured interviews and classroom teaching observation; the remaining four interviewees did not contribute classroom observation data, and two observed participants did not complete the interview. Because the qualitative subsample is small (eight interviews and six lessons), all qualitative material is treated as exploratory and is not used to support generalisable claims about pre-service TCFL teachers. Qualitative findings are reported throughout as themes described by these participants, not as population-level estimates. This overlap information is important for interpreting triangulation: for participants contributing both interview and observational data, we can cross-check self-reported subjective narratives against their real-world instructional behaviors; for participants contributing only one qualitative data source, evidence is interpreted with appropriate caution. Our integration follows a joint-display-inspired analytical workflow: quantitative group-level patterns, interview narrative excerpts and coded classroom behavioral records are systematically brought together during interpretation to build integrated understandings; due to journal page-limit constraints, a full standalone joint-display summary table is not included within the manuscript text. Single questionnaire research can only obtain self-reported subjective scores, while mixed methods combine objective classroom behavioral evidence and participants’ inner psychological narration to make the research conclusion more rigorous (Dong, 2023).

### 3.1 Overall explanatory sequential mixed-method research design

This design includes two successive research phases with integrated result interpretation. Phase One is cross-sectional quantitative survey, aiming to describe the imbalanced characteristics of pre-service TCFL’s seven-dimensional TPACK self-efficacy belief system, and test inter-group differences and dimensional correlations. Phase Two is qualitative supplementary research, using one-on-one interviews and classroom video coding to extract implicit affective moderators such as employment anxiety and cross-cultural teaching stress that cannot be captured by quantitative scales, and explain the psychological logic of significant or non-significant quantitative results. Single questionnaire research can only obtain self-reported subjective scores, while mixed methods combine objective classroom behavioral evidence and participants’ inner psychological narration to make the research conclusion more rigorous (Dong, 2023).

### 3.2 Research participants

Recruitment adopted convenience sampling combined with snowball sampling from four normal universities in Northeast China. A total of 202 valid questionnaires were recovered, with an effective recovery rate of 90.6%. All participants meet two unified screening criteria: native Mandarin speakers who passed Mandarin Level 2A, CET-6 or equivalent English proficiency, full-time pre-service students without formal full-time Chinese teaching posts. All participants’ teaching practice adopted online remote forms due to pandemic restrictions, unifying online teaching stress exposure to eliminate environmental confounding variables. Complete demographic statistics of all participants are presented, as shown in Table 2.

**Table 2:** Demographic distribution of 202 pre-service TCFL.

Demographic category	Subgroup	Number	Percentage (%)
Gender	Male	21	10.4
	Female	181	89.6
Age group	Under 22	17	8.4
	22–25	157	77.7
	26 and above	28	13.9
Academic stage	Undergraduate	30	14.9
	Master’s student	170	84.2
	Doctoral candidate	2	1.0
Undergraduate major	TCFL	141	69.8
	Foreign languages	29	14.4
	Chinese language and literature	21	10.4
	Other majors	11	5.4
Edtech training experience	Completed training	128	63.4
	No training	74	36.6
Teaching competition experience	Participated	108	53.5
	Never participated	94	46.5
Cross-cultural teaching practice	Have experience	149	73.8
	No experience	53	26.2
Teaching tenure (only for participants with practice)	<0.5 year	30	20.1
	0.5–1 year	62	41.6
	1–2 years	45	30.2
	>2 years	12	8.1

From the demographic data displayed in Table 2, multiple representative sample distribution features can be summarized from the perspective of motivational psychology research. First, the gender structure is extremely unbalanced: female participants account for 89.6% of the total sample, which conforms to the long-term enrollment characteristic of TCFL majors, yet the severe gender imbalance will reduce the statistical power of subsequent gender difference tests (Lee, 2020). Second, the age distribution is highly concentrated in the 22–25 age group (77.7%), which corresponds to the core training stage when pre-service teachers’ TPACK self-efficacy and motivational functioning are initially shaped, matching the core research objective of this paper. Third, master’s students constitute the absolute main body of the sample (84.2%), reflecting the mainstream training path for future Chinese language educators. Fourth, nearly 70% of participants majored in TCFL during undergraduate study, meaning most samples have systematic second-language pedagogy training and relatively stable core PCK pedagogical beliefs. Fifth, 73.8% of respondents accumulated online cross-cultural teaching practice experience, providing sufficient comparative samples to analyze the differentiated effects of teaching experience on teachers’ technology integration motivation.

### 3.3 Three standardized research instruments

#### 3.3.1 TPACK self-perceived competence questionnaire

This study was based on the TPACK self-assessment scale developed by Schmidt et al. (2009), and was adapted in combination with the characteristics of teaching Chinese as a foreign language and the latest research results to form the “Survey Scale for the Current Status of TPACK Levels of Pre-service Chinese Language Teachers”. The following section describes the systematic adaptation process in detail.

##### 3.3.1.1 Instrument adaptation process

The TPACK self-assessment scale developed by Schmidt et al. (2009) served as the foundation for our instrument. The original scale consists of 47 items distributed across seven dimensions (TK, CK, PK, PCK, TCK, TPK, and TPACK) and has been validated across multiple educational contexts. For the present study, we adapted this instrument specifically for the TCFL context through the following systematic procedures:

Step 1: Content Adaptation. We reviewed all 47 items from the original Schmidt et al. (2009) scale and identified items that required modification to reflect the unique characteristics of Chinese language teaching. The following types of modifications were made: (a) replacing general subject-content references with TCFL-specific content (e.g., replacing “mathematics” or “science” with “Chinese as a foreign language”); (b) adding TCFL-specific examples to item descriptions (e.g., “pinyin annotation software,” “Chinese language teaching databases,” “把字句 [ba-construction],” “声调 [tones]”); and (c) incorporating TCFL-specific teaching contexts (e.g., “online Chinese teaching,” “cross-cultural communication”).

Step 2: Expert Review. Three TCFL teacher educators with more than 5 years of experience in pre-service teacher training and two educational technology specialists reviewed the adapted items for content validity. Experts were asked to evaluate: (a) whether each item accurately reflected the corresponding TPACK dimension in the TCFL context; (b) whether the TCFL-specific examples were appropriate and representative; and (c) whether any important TCFL-specific knowledge or skills were missing. Based on expert feedback, we revised 12 items for clarity and added 3 items to better capture TCFL-specific technology integration practices (e.g., TCK4: “I am capable of teaching Chinese online”).

Step 3: Language Adaptation. The adapted instrument was developed in Chinese. To ensure conceptual equivalence with the original English version, we followed a translation and back-translation procedure. First, the adapted items were translated from English to Chinese by a bilingual researcher with expertise in TCFL. Second, a different bilingual researcher blind to the original English version back-translated the Chinese items into English. Third, the two English versions (original and back-translated) were compared by a panel of three researchers, and discrepancies were resolved through discussion. This process ensured that the Chinese items retained the conceptual meaning of the original items while being linguistically and culturally appropriate for Chinese-speaking pre-service TCFL teachers.

Step 4: Item Reduction and Refinement. The original Schmidt et al. (2009) scale contains 47 items. Based on expert review and the TCFL-specific adaptations, we reduced the total to 38 items. The rationale for item reduction was threefold: (a) some original items were not applicable to the TCFL context (e.g., items specific to mathematics or science teaching); (b) some items were redundant after the TCFL-specific adaptations; and (c) we aimed to balance the number of items across dimensions while ensuring adequate coverage of each construct. The final item allocation across dimensions is presented in Table 3. The reduction from 47 to 38 items was also informed by the pilot study (see Step 5 below), in which items with low item-total correlations or ambiguous wording were removed or revised.

**Table 3:** Item allocation of seven TPACK self-perceived competence dimensions.

Dimension full name	Abbreviation	Number of items	Core measurement connotation
Technological knowledge self-perceived competence	TK	6	Confidence in operating and troubleshooting Chinese teaching digital tools
Content knowledge self-perceived competence	CK	3	Confidence in mastering Chinese linguistics and intercultural disciplinary knowledge
Pedagogical knowledge self-perceived competence	PK	8	Confidence in diversified second-language teaching strategies
Pedagogical content knowledge self-perceived competence	PCK	5	Confidence in matching teaching methods with Chinese knowledge points
Technological content knowledge self-perceived competence	TCK	4	Confidence in using digital tools to display Chinese characters and tones
Technological pedagogical knowledge self-perceived competence	TPK	5	Confidence in optimizing classroom activities via digital resources
Overall TPACK integrated self-perceived competence	TPACK	7	Comprehensive confidence in complete digital Chinese lesson design

Step 5: Pilot Testing. A pilot study with 47 pre-service TCFL teachers was conducted to evaluate the clarity, relevance, and psychometric properties of the adapted items. Based on pilot results, we revised ambiguous items and confirmed that all items were understandable and relevant to the target population. The final 38-item instrument (see Appendix A for the complete scale) was used for formal data collection.

The questionnaire adopts a five-point Likert scoring system (1 = completely inconsistent, 5 = completely consistent), with a total of 38 formal measurement items corresponding to the seven psychological self-perceived competence constructs illustrated in Table 1 of the Literature Review chapter. The number of measurement items assigned to each self-perceived competence dimension and their core psychological measurement connotations are summarized in Table 3, as shown below.

Table 3 presents the rational layout of measurement items across seven self-perceived competence dimensions. The PK dimension contains the largest number of items (8), as pedagogical belief constitutes the core psychological foundation of sustained intrinsic teaching motivation and requires more indicators to capture subtle differences in self-confidence (Scherer et al., 2019). The overall integrated TPACK dimension includes seven items to reflect multi-layered psychological judgment of integrated teaching design. In contrast, the CK dimension only uses three items, as all participants received standardized Chinese disciplinary training, leading to narrow individual gaps in content belief. The item count of each dimension matches the complexity of its corresponding psychological construct, ensuring balanced measurement weight for all seven types of technology integration beliefs.

As described in Step 5 above, the pre-test with 47 participants was carried out to eliminate ambiguous and confusing items. Internal-consistency and factor-analytic suitability inspections were conducted on all 202 valid formal samples; the overall psychometric indicators of the scale are displayed in Table 4.

**Table 4:** Overall internal consistency and factor-analytic suitability inspection of the TPACK self-perceived competence questionnaire.

Inspection index	Test value	Criterion standard
Overall Cronbach’s alpha	0.969	>0.8, excellent internal consistency reliability
Global KMO value	0.951	>0.7, suitable for exploratory factor analysis
Bartlett’s sphericity test	*χ*^2^ = 5320.495, df = 702, *p* < 0.001	Extremely statistically significant

The overall Cronbach’s Alpha coefficient reaches 0.969, far exceeding the 0.8 threshold required for educational psychological measurement, demonstrating excellent internal consistency among questionnaire items. It should be noted that high internal consistency may also stem from item redundancy or high inter-item correlation and cannot be interpreted as construct validity evidence for the hypothesized seven-factor structure. The global KMO value of 0.951 indicates satisfactory sampling adequacy for potential factor-analytic procedures. The highly significant Bartlett’s Sphericity test result indicates that the correlation matrix is not an identity matrix, satisfying the prerequisite conditions for conducting factor analysis, but it does not by itself confirm the number of latent factors or the rationality of our theoretically-derived dimensional division. Detailed reliability and validity indicators for each independent self-perceived competence sub-dimension are listed in Table 5.

**Table 5:** Internal consistency and factor-analytic suitability for each TPACK self-perceived competence sub-dimension.

Dimension	Cronbach’s alpha	KMO	Bartlett significance
TK	0.812	0.819	*p* < 0.001
CK	0.827	0.711	*p* < 0.001
PK	0.902	0.913	*p* < 0.001
PCK	0.876	0.838	*p* < 0.001
TCK	0.831	0.774	*p* < 0.001
TPK	0.864	0.858	*p* < 0.001
TPACK	0.895	0.906	*p* < 0.001

All sub-dimension Cronbach’s Alpha coefficients range from 0.812 to 0.902, all meeting the satisfactory internal consistency standard above 0.8. Among all dimensions, the PK dimension owns the highest Alpha (0.902) and KMO value (0.913), which corresponds to the item allocation rule in Table 3 and suggests that pedagogical self-perceived competence shows comparatively stable internal responses in this sample. Even the lowest KMO value (CK = 0.711) still exceeds the critical value of 0.7, demonstrating each set of sub-dimension items satisfies basic prerequisites for factor analysis. Statistically significant Bartlett’s sphericity tests indicate that sub-dimension correlation matrices deviate from identity matrices. These statistics only provide evidence for internal consistency and factor-analysis suitability; they do not offer empirical proof that these seven theoretical sub-dimensions are empirically independent, discriminant, or represent unified latent constructs. The item allocation of each dimension is displayed in Table 3. The complete full-text items of this seven-dimensional self-perceived competence measurement scale are provided in Appendix A.

#### 3.3.2 Motivational psychology-oriented semi-structured interview outline

The semi-structured interview outline was deductive and theory-driven, developed drawing on Self-Determination Theory and prior TPACK motivational literature. Several interview prompts reference pre-specified theoretical constructs (e.g., basic psychological-need satisfaction, extrinsic motivation, psychological depletion). Therefore, resulting qualitative themes cannot be treated as entirely unanticipated discoveries emerging purely inductively from participant responses. Instead, interviews explored participants’ lived experiences and subjective perceptions in relation to these theoretically relevant constructs.

The design logic of this interview outline fully abandons single technical and teaching discussion content, and all ten core interview questions revolve around the core themes of this Research Topic: motivational functioning, internal psychological processes, pedagogical beliefs and professional well-being. The overall interview design draws on the mature qualitative research paradigm of Tondeur et al. (2017), aiming to dig out perceived contextual barriers and exploratory affective qualitative themes that cannot be captured by quantitative questionnaires, such as intrinsic/extrinsic motivation differences, technical anxiety, employment pressure and cross-cultural teaching psychological stress. Full core interview questions are listed as follows:

1. What internal and external factors will raise or reduce your intrinsic motivation to independently design digital Chinese teaching lessons? How will your overall technology integration self-efficacy change accordingly?
2. What kinds of cross-cultural online teaching difficulties will bring you psychological pressure and technical anxiety? How do these negative psychological states affect your long-term willingness to engage in Chinese teaching?
3. How do educational technology training, teaching skills competitions and internship practice change your satisfaction of autonomy, competence and relatedness psychological needs? Will these changes affect your long-term professional well-being?
4. Employment competition belongs to typical extrinsic motivation. Will the pressure of job hunting force you to learn digital teaching tools actively? Will long-term dependence on such external motivation lead to psychological depletion?
5. What obvious psychological differences exist in your self-confidence when you only use digital tools to display knowledge versus integrating technology, teaching methods and Chinese content to design complete lessons?
6. What feedback from tutors and peer students can effectively consolidate your core pedagogical beliefs and activate intrinsic digital teaching motivation?
7. What psychological gains or emotional exhaustion will you experience after participating in Chinese teaching skills competitions?
8. How will long-term continuous cross-cultural teaching practice reshape your PCK and TPK core motivational beliefs?
9. What Chinese-specific digital tools can significantly improve your teaching self-efficacy? What technical operations will reduce your self-confidence and trigger technical anxiety?
10. What targeted psychological training and intervention modes can better improve your intrinsic digital teaching motivation and protect your long-term professional well-being?

Recruitment adopted purposive sampling from valid questionnaire respondents. A total of 8 participants with different age, major, training and teaching tenure backgrounds were selected for one-on-one online interviews, with total recording time of 80 min. All audio was transcribed word-for-word for thematic coding. The basic information table of eight interviewees is not displayed in the main text, see Appendix B. For overlap between interview participants and classroom-observation participants, please see Section 3.1.

#### 3.3.3 Classroom motivational behavior coding scheme

The coding framework is adapted from Harris et al. (2010), converting all classroom teaching behaviors into external manifestations of latent TPACK self-efficacy psychology. Six complete online Chinese teaching videos were coded independently by two researchers; inter-coder reliability κ = 0.83, reaching the standard of qualitative thematic analysis (Braun and Clarke, 2006). The standardized coding classification criteria are presented in Table 6.

**Table 6:** Observable classroom instructional-behavior coding scheme for TPACK-related teaching actions.

Code	Observable instructional behavior	Specific coding standard
TK	Technology-troubleshooting actions	Hesitant operation, repeated debugging of digital tools during class sessions
CK	Disciplinary-content exposition	Independent explanation of Chinese-language subject knowledge without digital-tool support
PK	Pedagogical-strategy deployment	Active deployment of diversified interactive teaching and classroom-management strategies
PCK	Pedagogy-content matching practice	Actions that align instructional-teaching methods with Chinese-subject knowledge points
TCK	Technology-supported content presentation	Employing digital tools primarily for static presentation of Chinese-language subject content
TPK	Technology-pedagogy adaptation practice	Adjusting and adapting digital resources to enhance student classroom engagement and instructional delivery
TPACK	Integrated technology-pedagogy-content practice	Lesson segments featuring simultaneous integration of technology, pedagogy and Chinese-subject-content elements
N	Neutral off-task content	Casual dialog and utterances unrelated to instructional activities

Table 6 captures only directly-observable classroom instructional behaviors, without embedding a-priori motivational-psychological interpretations within coding categories. Inter-coder reliability κ = 0.83 demonstrates consistent rater agreement for classifying visible teaching actions; this reliability index does not validate any underlying latent motivational or self-efficacy-related psychological constructs. Motivational-psychological inferences are not derived directly from behavioral codes alone. Instead, interpretations regarding self-perceived-competence and motivational functioning are generated in the analysis phase through triangulation of coded observational records, questionnaire-scale results and semi-structured-interview participant narratives. The complete original classroom coding form is stored in Appendix C.

### 3.4 Full research implementation procedures

#### 3.4.1 Pilot pre-experiment

Before formal large-scale data collection, 47 pre-service students were invited to complete the TPACK questionnaire pre-test. Normality inspection and internal-consistency analysis were conducted on pre-test data. Based on pre-test statistical outputs and participants’ written feedback, we revised wording for several semantically ambiguous items, removed items demonstrating low item-total correlation, and fine-tuned Likert-scale response descriptions. After these revisions, all indicators met acceptable standards before formal distribution.

#### 3.4.2 Formal quantitative data collection

The formal questionnaire was distributed via university email in September 2022, with research purpose, anonymity commitment and filling guidance attached to the email. After collecting 202 valid responses, stratified purposive sampling was used to select 8 interview participants and 6 observation objects from all respondents.

#### 3.4.3 Qualitative data collection process

Online one-on-one interviews were scheduled after obtaining participants’ written signed informed consent, with whole-process audio recording. Six online teaching videos submitted by participants were collected as classroom observation materials; all teaching scenes are remote online modes formed under pandemic restrictions.

### 3.5 Data analysis steps

#### 3.5.1 Quantitative statistical analysis

All valid questionnaire data were imported into SPSS 26.0 software for standardized statistical processing (Dong, 2023). The analytical workflow was as follows: First, descriptive statistics including mean, standard deviation, median, minimum and maximum values were calculated to summarize self-perceived competence across each TPACK dimension. Second, two-tailed Pearson correlation analysis was conducted to examine inter-construct associations among the seven TPACK belief components. Third, Shapiro–Wilk normality tests and Levene homogeneity-of-variance tests were performed prior to every inter-group comparison, and concrete test statistics are reported in-text and in supplementary tables. For binary categorical grouping variables (gender, technical-training experience, competition experience, teaching experience), independent-sample t-tests were applied. Multi-category variables satisfying homogeneity of variance were analysed via one-way ANOVA with Bonferroni-adjusted post-hoc tests; groups violating homogeneity assumptions were analysed using non-parametric Kruskal-Wallis tests.

All valid questionnaire data were analysed in SPSS 26.0 (Dong, 2023). First, we computed descriptive statistics (mean, standard deviation, median, minimum and maximum) for each TPACK dimension. Second, we ran two-tailed Pearson correlations among the seven dimensions. Third, we ran inter-group comparisons. Before each comparison we tested normality (Shapiro–Wilk) and homogeneity of variance (Levene). We used independent-sample t-tests for binary grouping variables (gender, technical-training experience, competition experience, teaching experience). We used one-way ANOVA with Bonferroni-adjusted post-hoc tests for multi-category variables that met the homogeneity assumption, and Kruskal-Wallis tests for those that did not. We ran many univariate comparisons across seven outcome dimensions. To limit false positives, we grouped them into test families and applied Bonferroni-corrected thresholds. The nominal significance level was α = 0.05, and we report corrected criteria alongside each test family. Results with 0.04 ≤ *p* < 0.05 are treated as tentative, not as firm conclusions. We report effect sizes (Cohen’s d, η^2^, ε^2^) with 95% confidence intervals, together with test statistics and degrees of freedom.

Importantly, this study relies on cross-sectional bivariate group comparisons and does not implement multivariate predictive modeling. Accordingly, only associational interpretations are presented throughout the manuscript; we avoid causal claims about “effects” or “influences.” Inferential results derived from very small subgroups (e.g., doctoral candidates n = 2, participants aged under 22 years n = 17, male participants n = 21) possess limited statistical power and should be regarded as exploratory descriptive observations requiring replication in larger, better-balanced participant samples.

#### 3.5.2 Qualitative thematic analysis

All interview transcripts and classroom coding records were analysed using reflexive thematic analysis following Braun and Clarke (2006). This approach follows recursive analytical phases: familiarization with raw materials, generation of initial descriptive codes, development of candidate themes, iterative theme review, theme definition, and analytical write-up. Two independent researchers familiarized themselves with transcripts, generated initial descriptive codes, then developed and reviewed candidate themes. Disagreements on theme boundaries were resolved through discussion with a third researcher.

Across the later subset of eight interview transcripts, no additional high-order affective themes emerged during analytical review. Given the relatively small qualitative corpus (eight interviews, total recording duration of 80 min), we do not claim full theoretical saturation in the grounded-theory sense. This observation only indicates diminishing arrival of new high-order themes within this specific participant sample.

For classroom video analysis, two researchers coded separately based on Table 6; disagreements were resolved through third-party review, with inter-coder reliability κ = 0.83. Detailed information of the six observed teaching videos is not presented in the main text, and can be checked in Appendix C.

Following this recursive coding workflow, four candidate core axial themes were anticipated to emerge from the dataset: imbalanced TPACK belief profiles, intrinsic-extrinsic motivational regulation orientations, positive experiential motivational drivers, and negative affective teaching-related stressors. These potential thematic categories guided our iterative theme-reviewing procedure, while we remained open to unanticipated themes from participant raw narratives (Figure 3).

**Figure 3:**
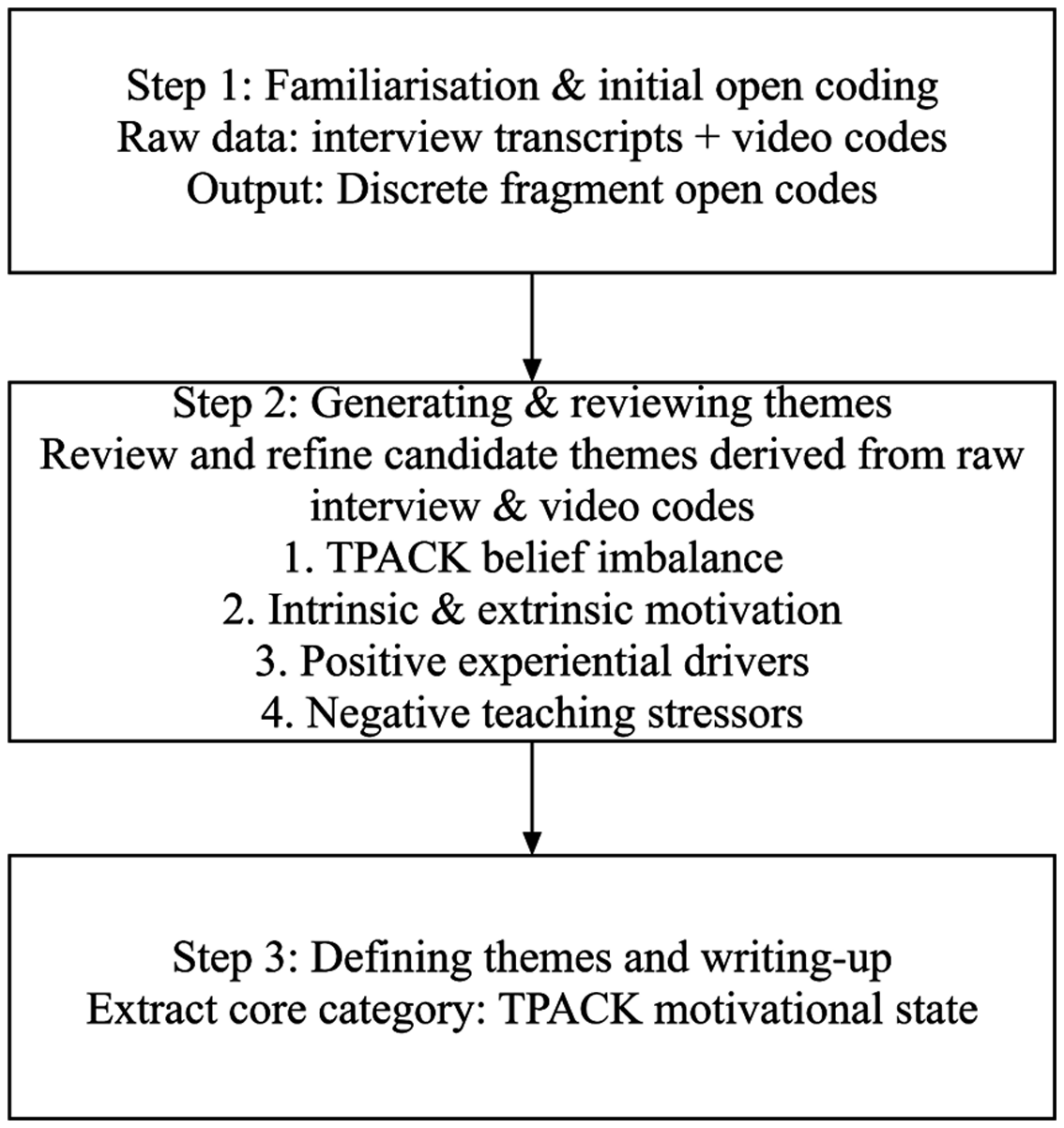
Coding workflow for reflexive thematic analysis (Braun and Clarke, 2006) for interview and classroom-coding qualitative data.

## 4 Results

### 4.1 Descriptive statistical results of seven TPACK self-perceived competence dimensions

Descriptive statistics including sample size, minimum value, maximum value, mean score and standard deviation of each TPACK dimension are presented in Table 7, which intuitively reflects the imbalanced distribution of pre-service TCFL’s technology integration belief system (Scherer et al., 2019).

**Table 7:** Overall descriptive statistics of seven TPACK self-perceived competence dimensions.

Dimension	*N*	Minimum	Maximum	Mean	Standard deviation	Median
TK	202	2.33	5.00	3.6815	0.57859	3.667
CK	202	1.00	5.00	3.8053	0.64606	4.000
PK	202	2.00	5.00	3.8038	0.59102	3.875
PCK	202	2.00	5.00	3.7950	0.55051	3.900
TCK	202	2.00	5.00	3.8255	0.62855	4.000
TPK	202	2.00	5.00	3.7881	0.63234	3.800
TPACK	202	2.00	5.00	3.7433	0.57521	3.857

All dimensions adopt a five-point Likert scoring system, and the average scores of all seven dimensions range between 3.68 and 3.83, indicating that participants’ self-perceived TPACK competence, which we interpret as an indicator of technology-integration self-efficacy beliefs, is at the medium-upper level, consistent with the basic data characteristics of language pre-service teacher samples in previous research (Liu and Wang, 2022). Differences between dimensions were small in absolute terms. All seven means fall within a narrow band (3.68–3.83): a spread of 0.15 points on a five-point scale, against standard deviations of 0.55–0.65 that are large relative to that spread. We therefore describe the pattern descriptively rather than as a statistically significant imbalance, because no formal test comparing the seven dimension means (for example, a repeated-measures ANOVA with sphericity correction) was conducted. With that caution stated, TK obtained the lowest mean (M = 3.68) and TCK the highest (M = 3.83). The TK mean suggests comparatively lower self-perceived competence in complex digital tool operation and technical troubleshooting, a pattern that is consistent with, but does not demonstrate, the theoretical possibility that competence-related experiences in the technical domain are less well supported (Dong, 2023). By contrast, the TCK dimension reaches the highest mean value (M = 3.83). Combined with interview data, this high score originates from the large-scale mandatory online teaching practice triggered by public health events, and the motivation behind this confidence belongs to short-term extrinsic motivation rather than spontaneous instructional interest (Tondeur et al., 2017). The overall integrated TPACK dimension has the second-lowest mean score (M = 3.74), demonstrating that although pre-service teachers possess independent confidence in single technical or disciplinary content tasks, they lack stable psychological support for the three-way integrated design of technology, pedagogy and Chinese subject content (Voogt et al., 2016). Further thematic extraction from interview transcripts verified this dimensional gap. Multiple interviewees mentioned that they could proficiently use software to display Chinese characters and phonetic symbols, but felt confused when designing complete online teaching units combining teaching methods and interactive activities. As Participant F stated: “If it involves advanced technical operation and whole-lesson integrated design, I will lack confidence and hesitate to try independent creation”. This imbalanced profile low TK confidence coupled with high TCK confidence suggests a pattern of externally driven motivational functioning: trainees feel competent when technology use is mandated and scaffolded by external requirements (online teaching mandates), yet lack the autonomous confidence to voluntarily explore or innovate with technology, a hallmark of intrinsic motivation deficit (Ryan and Deci, 2020).

### 4.2 Pearson correlation analysis of TPACK self-perceived competence dimensions

Two-tailed Pearson correlation analysis was conducted on all seven TPACK dimensions to clarify the internal motivational-cognitive linkage among different self-efficacy beliefs, and the complete two-tailed Pearson correlation matrix of seven TPACK dimensions is displayed in Table 8.

**Table 8:** Pearson correlation matrix of seven TPACK self-perceived competence dimensions.

Correlation							
Project	TK	CK	PK	PCK	TCK	TPK	TPACK
TK	1						
CK	0.519^**^	1					
PK	0.578^**^	0.751^**^	1				
PCK	0.545^**^	0.675^**^	0.853^**^	1			
TCK	0.578^**^	0.657^**^	0.820^**^	0.783^**^	1		
TPK	0.580^**^	0.624^**^	0.771^**^	0.768^**^	0.786^**^	1	
TPACK	0.587^**^	0.639^**^	0.781^**^	0.784^**^	0.769^**^	0.835^**^	1

^**^At the 0.01 level (2-tailed), there is a significant correlation. ^**^p < 0.01, two-tailed significance test.

All pairwise correlation coefficients among seven dimensions reach an extremely significant positive level (*p* < 0.01), suggesting that all types of technology integration self-efficacy beliefs form an interconnected whole motivational cognitive system; the deficiency of any single belief will restrain the overall integrated teaching motivation (Mishra and Koehler, 2006). From the correlation strength between each sub-dimension and the overall TPACK construct (referring to the seven-item integrated TPACK sub-dimension, not an aggregate total score across all 38 items): TPK (*r* = 0.835) and PCK (*r* = 0.784) show the strongest correlation coefficients, while the correlation between TK and overall TPACK is the weakest (*r* = 0.587). These pairwise correlation magnitudes reflect only linear associational strength among self-perceived competence dimensions. Zero-order correlations alone cannot establish structural centrality, causal priority, or causal influence between constructs. This pattern nevertheless runs counter to techno-centric assumptions that “mastering digital tools can realize high-quality integrated teaching” (Scherer et al., 2019). Pure technical self-perceived competence shows comparatively weaker linkage with integrated TPACK. Theoretical reasoning regarding potential “core psychological hubs” is reserved for the Discussion section, as correlation results cannot empirically validate such structural claims. The correlation difference also provides empirical support for the training optimization direction in the discussion chapter: pre-service teacher education should prioritize the cultivation of pedagogical integration ability rather than isolated technical operation training (Tondeur et al., 2017). Classroom behavioral coding results further echoed this correlation rule: more than 70% of teaching behaviors recorded in videos belonged to PCK and TPK integrated operation, while independent TK technical debugging behaviors appeared rarely in complete teaching sessions. The finding that PCK and TPK correlate most strongly with overall TPACK, while TK correlates least, challenges techno-centric training paradigms and supports a motivational hub model: sustainable intrinsic motivation for technology-integrated teaching hinges on pedagogical confidence—which satisfies autonomy and relatedness needs rather than on isolated technical proficiency, which primarily addresses superficial competence needs without fostering deeper psychological ownership.

### 4.3 Inter-group difference test results of influencing factors

Before inter-group comparison, Shapiro–Wilk normality test and Levene homogeneity of variance test were carried out for each dimensional data. For groups conforming to normal distribution and equal variance, independent sample t-test or one-way ANOVA was adopted; for data violating the two premises, Kruskal-Wallis non-parametric test was used, with the unified significance threshold set as *p* < 0.05. All grouping variables are divided into two categories: static demographic factors and dynamic experiential factors. Full demographic, descriptive, correlation and group-difference statistics are provided in Supplementary Tables S1–S4.

#### 4.3.1 Demographic variable differences

Gender. Independent sample t-test results indicated that all seven TPACK dimensions had no significant gender difference (all *p* > 0.05). Taking the overall TPACK dimension as an example: male M = 3.77, SD = 0.61; female M = 3.74, SD = 0.57; *t* = 0.901, *p* = 0.369. Consistent with cross-cultural comparative research conclusions (Lee, 2020), gender does not produce inherent disparities in technology integration motivation and self-confidence of language pre-service teachers. The non-significant gender effects, despite the severely imbalanced sample, are consistent with cross-cultural motivational research (Lee, 2020), suggesting that motivational belief formation depends more on experiential mastery than on demographic traits. However, it is necessary to note the serious gender imbalance of this sample (only 21 male participants), which reduces the statistical test power of gender comparison, and the conclusion needs to be verified by a more balanced sample in follow-up research. Interview data also supported this result: both male and female interviewees stated that their confidence in digital teaching mainly depended on teaching practice experience rather than gender characteristics.

Age grouping. Participants were divided into three age cohorts: under 22 years old (*n* = 17), 22–25 years old (*n* = 157), and 26 years old and above (*n* = 28). Shapiro–Wilk tests confirmed approximate normality for all outcome variables and Levene’s tests indicated homogeneity of variance. One-way ANOVA revealed significant group-level associations for PK (F[2, 199] = 3.08, *p* = 0.028, *η*^2^ = 0.031, 95% CI [0.001, 0.080]) and TCK (F [2, 199] = 3.79, *p* = 0.011, *η*^2^ = 0.038, 95% CI [0.003, 0.089]). Bonferroni-corrected post-hoc comparisons indicated that participants younger than 22 years reported lower self-perceived PK and TCK competence relative to the other two age cohorts. Note: the under-22 subgroup is small (*n* = 17); these borderline results should be regarded as tentative suggestive associational patterns rather than definitive conclusions and await replication in larger samples.

Undergraduate major background. One-way ANOVA found that only the PC dimension had significant inter-group differences (*F* = 2.721, *p* = 0.046). Multiple comparison results showed that pre-service teachers with Teaching Chinese as a Foreign Language and foreign language undergraduate backgrounds had significantly higher PCK self-efficacy than students from irrelevant majors (Castéra et al., 2020). Systematic second language pedagogy training in undergraduate stages helps students form stable core pedagogical beliefs, while non-language majors lack long-term disciplinary teaching accumulation, resulting in insufficient matching confidence between teaching methods and Chinese knowledge points. No significant differences existed in other dimensions.

Academic stage (undergraduate/master/doctor). Kruskal-Wallis non-parametric test results showed that all seven TPACK dimensions had no significant differences among different academic stages (all *p* > 0.05). Educational attainment cannot become an effective predictor of technology integration self-efficacy; the formation of motivational beliefs mainly relies on hands-on teaching experience rather than academic credentials (Yildiz Durak et al., 2023). The sample was dominated by master’s students (84.2%), which limited the differentiation effect of academic grouping to a certain extent.

#### 4.3.2 Experiential variable differences

Educational technology training experience. Independent-sample t-test showed that participants who had completed educational-technology-related training reported significantly higher self-perceived TCK competence compared with participants without such training experience (*t* [200] = 2.61, *p* = 0.010, Cohen’s *d* = 0.37, 95% CI [0.09, 0.66]), while no statistically significant associations were observed for PCK, TPK or overall integrated TPACK dimensions. The significant TCK advantage among trainees who received edtech training, unaccompanied by gains in PCK or TPK, illustrates the limitation of competence-only interventions: tool-focused training satisfies the need for skill acquisition but fails to address autonomy and relatedness needs, thereby generating only short-term extrinsic motivation (Voogt et al., 2016). As pointed out by Voogt et al. (2016), most university technical training adopts tool-centered modes, only satisfying superficial competence needs of operating software to display content, without providing independent curriculum design space to meet autonomy needs, so it can only generate short-term extrinsic motivation and cannot cultivate long-term integrated teaching willingness. Interview participants who only took basic tool courses repeatedly mentioned that they learned how to make courseware but could not flexibly match teaching methods with digital resources.

Chinese teaching skills competition experience. Participants with competition experience had significantly higher PK dimension self-efficacy (*t* = 2.060, *p* = 0.041). Teaching competitions bring one-time achievement feedback to satisfy temporary competence needs, temporarily enhancing teachers’ pedagogical design confidence and competitive extrinsic motivation (Zou et al., 2022). However, the short-term stimulation effect cannot be transformed into lasting intrinsic motivation without long-term continuous teaching practice accumulation.

Participants with cross-cultural teaching-practice experience reported significantly higher self-perceived scores across PK, PCK, TCK and overall integrated TPACK dimensions compared to participants without such experience. Further Kruskal-Wallis test of teaching tenure subgroups (<0.5 year, 0.5–1 year, 1–2 years,>2 years) revealed that participants with practice duration exceeding six-months reported higher overall self-perceived TPACK competence relative to those with less-than-six-month practice. This is merely a cross-sectional associational pattern observed within our sample. Our snapshot data cannot demonstrate that practice duration itself produces motivational stabilization after six-months; pre-existing individual differences among participants may also explain this observed grouping difference. The six-month cut-off only serves as an exploratory descriptive sample split rather than an empirically validated critical threshold for motivational change.

Different teaching internship identities. Kruskal-Wallis test results indicated that training institution teachers, Confucius Institute volunteers and university student instructors had no significant TPACK dimensional differences (all *p* > 0.05). The type of internship platform will not change teachers’ core motivational beliefs, as long as continuous front-line teaching accumulation can satisfy three basic psychological needs.

### 4.4 Supplementary qualitative evidence from interviews and classroom coding

Through three-stage thematic analysis (open-axial-selective coding), four core axial themes were extracted from interview transcripts and classroom behavioral coding records: (1) Imbalanced TPACK belief profiles across seven dimensions; (2) Intrinsic versus extrinsic motivational regulation orientations; (3) Positive motivational drivers from experiential factors (training, competition, cross-cultural teaching practice); (4) Negative affective stressors including employment-market-related pressure and cross-cultural online-teaching stress. Quantitative statistical results reflect overall group trends, while semi-structured interview transcripts and classroom observational coding data help to identify implicit perceived contextual barriers and exploratory qualitative affective themes that cannot be captured by questionnaires (Tondeur et al., 2017). Because this qualitative subsample comprises only eight interviews and six lessons, these themes are reported as exploratory descriptions of what these participants reported, and are not generalized to pre-service TCFL teachers as a population. When conducting triangulation, we distinguish participants who contributed both interview and classroom observation materials versus those who provided only one qualitative data source; evidence from multi source participants enables direct cross validation between subjective accounts and observable teaching behaviors. Two core inhibiting psychological factors were summarized through thematic coding:

Regarding employment-competition-related pressure (perceived contextual barrier):

“I mainly learn digital teaching tools because I fear being eliminated in job-hunting; I seldom take the initiative to explore interesting Chinese teaching design out of genuine interest.” (Participant B)

Regarding cross-cultural online-teaching-related stress:

“Time-zone differences and unstable network connections often ruin my online Chinese teaching attempts; repeated failures make me less willing to try integrated digital-lesson design.” (Participant E)

First, employment competition pressure. Several of the eight interviewees stated that they learned online teaching tools only to meet the entry requirements of Chinese education positions, belonging to single extrinsic motivation driven by survival pressure. Long-term over-reliance on such external driving force will produce chronic emotional depletion, reduce their spontaneous interest in curriculum design, and damage long-term professional well-being (Klassen and Chiu, 2010).

Second, cross-cultural online teaching stress including time zone gaps, unstable network signals and cross-cultural communication barriers. Frequent teaching failures caused by environmental constraints will trigger continuous negative emotional arousal, lower teachers’ overall TPACK self-confidence, and suppress their willingness of autonomous integrated teaching design (Dong, 2023).

Classroom observational coding documented the distribution of observable instructional behaviors across lesson segments: standalone TK-related technology-troubleshooting actions occurred infrequently, whereas PCK- and TPK-type instructional practices occupied large proportions of coded teaching time. This behavioral pattern constitutes descriptive observational evidence. We report only the distribution of observed behaviors here. Interpretations of these behavioral patterns are developed in the Discussion (Section 5.2), where they are triangulated with questionnaire and interview evidence. Detailed classroom video information of six observation objects is attached to Appendix C for reference.

## 5 Discussion

Interpretive Note on the Use of Self-Determination Theory. Before discussing the findings, we state the status of Self-Determination Theory (SDT) in this study. SDT is not tested here. We administered no measure of intrinsic or extrinsic motivation, of autonomy, competence or relatedness need satisfaction, or of motivational regulation. Where SDT vocabulary appears below, it is used only as an interpretive lens: a way of describing patterns in self-perceived TPACK competence that is drawn from an established theory. Every SDT-based statement should therefore be read as a theoretical interpretation of observed patterns, not as evidence that basic psychological needs were satisfied or frustrated. Readers should not infer from this discussion that motivational mechanisms have been demonstrated.

This study draws on Bandura’s social cognitive theory and autonomy theory, and analyzes the quantitative and qualitative research results presented in the study within the current research context of TPACK and the functions of teachers’ motivation. Two scope conditions apply to everything that follows. First, all quantitative results are cross-sectional and correlational. Second, all qualitative results come from eight interviews and six observed lessons, so they are treated as exploratory and are not generalized beyond this sample. The study examines the potential psychological mechanisms underlying the observed statistical patterns and clarifies the theoretical and practical advancements brought about by this mixed-method research focusing on Chinese as a foreign language teacher (TCFL) during the pre-service stage.

Interpretive Note on the Self-Efficacy Framework. Throughout this discussion, we interpret differences in self-perceived TPACK scores as indicators of differences in technology-integration self-efficacy beliefs. This interpretation is grounded in Bandura's (1997) social cognitive theory, which posits that self-efficacy beliefs are formed through four primary sources: mastery experiences, vicarious experiences, verbal persuasion, and emotional arousal. The TPACK self-report items capture individuals’ perceived capability to execute technology-integrated teaching tasks—a form of self-judgment that closely approximates Bandurian self-efficacy. However, we acknowledge that our instrument measures perceived competence (a broader self-assessment) rather than task-specific self-efficacy (a more narrowly defined construct). The conceptual distinction between perceived competence and self-efficacy is important: self-efficacy refers specifically to judgments of capability to organize and execute courses of action required to attain designated types of performances (Bandura, 1997), whereas perceived competence is a more general self-evaluation of knowledge and ability. We interpret our TPACK scores as indicators of technology-integration self-efficacy beliefs, recognizing this as a theoretically grounded interpretation rather than a claim of psychometric equivalence. This interpretive approach is consistent with a growing body of TPACK research that treats self-report measures as proxies for self-efficacy beliefs (Scherer et al., 2019; Dong, 2023).

### 5.1 Explanation of imbalanced TPACK self-efficacy dimensional profiles (RQ1)

The dimensional distribution reflected in Table 7 provides a descriptive basis for examining pre-service teachers’ differentiated self-perceived TPACK competence, which we interpret as reflecting their technology-integration self-efficacy beliefs. The descriptive statistics displayed in Table 7 reveal medium-to-high overall TPACK self-efficacy across participants (M range: 3.68–3.83), alongside prominent cross-dimensional disparities that can be illuminated through Bandura’s (1997) four pathways of self-efficacy formation. TK registers the lowest mean score (M = 3.68), signifying limited confidence in advanced technical operations and classroom troubleshooting. As Luo (2022) outlined in a scoping review of TCFL TPACK scholarship, mainstream pre-service training programs only deliver introductory software tutorials without scenario-based technical training tailored to Chinese character and tone instruction. Sustained deficits in mastery experience deprive learners of consistent positive feedback, leaving their competence needs unmet and dampening intrinsic enthusiasm for innovative digital lesson design (Ryan and Deci, 2020).

By contrast, TCK attains the highest average value (M = 3.83). This pattern stems from the universal shift to remote instruction driven by public health restrictions, which forced trainees to routinely deploy digital resources for presenting linguistic and cultural content (Tondeur et al., 2017). Interview extracts further clarify that elevated TCK confidence arises primarily from extrinsic pressures tied to graduate employment criteria, rather than organic instructional interest, rendering this confidence fragile and task-bound (Scherer et al., 2019). The composite TPACK dimension yields the second-lowest average (M = 3.74), confirming that isolated mastery of technology or subject content fails to translate into integrated lesson-planning capacity—a consistent limitation documented across cohorts of language pre-service educators (Voogt et al., 2016).

Notably, this study’s TCK-centric profile diverges from English language teaching research where TPK dominates self-efficacy ratings (Liu and Wang, 2022). Such divergence underscores the contextual specificity of TPACK belief structures: written Chinese’s logographic system and tonal phonetics demand specialized digital visualization tools, making content-technology alignment a daily instructional priority for TCFL practitioners. Variations across linguistic teaching contexts demonstrate that TPACK dimensional hierarchies cannot be generalized without disciplinary calibration (Zou et al., 2022).

Beyond descriptive interpretation, the observed dimensional imbalance reveals a dysfunctional motivational profile with implications for professional well-being. Low TK mean scores point toward frustrated competence-related psychological needs within the technical domain. According to Self-Determination Theory, this kind of unmet need represents a psychological state that may precede disengagement and avoidance-oriented behavioral patterns (Ryan and Deci, 2020). Concurrently, the elevated TCK scores, tied as they are to external mandates rather than intrinsic interest, reflect externally regulated motivation the most controlled and least autonomous form of behavioral regulation. According to SDT’s organismic integration theory (Ryan and Deci, 2020), externally regulated motivation, while effective for short-term task compliance, fails to sustain engagement over time and leaves individuals vulnerable to psychological depletion when external contingencies are removed. Thus, the TPACK profile documented here is not merely an educational competency gap; it is a motivational risk profile that, if unaddressed, may accelerate burnout and undermine career persistence.

### 5.2 Core motivational hub mechanism reflected by dimensional correlations (RQ2)

The significant inter-dimensional correlation results shown in Table 8 (presented within the main manuscript body) verify the integrated nature of the TPACK framework, and the classroom coding rules summarized in Table 6 (also in the main manuscript body) further support the dual-core hub mechanism of PCK and TPK. All pairwise correlations in Table 8 register statistically significant positive associations at the *p* < 0.01 threshold, corroborating the interconnected, holistic nature of the TPACK framework conceptualized by Mishra and Koehler (2006). TPK, (*r* = 0.835) and PCK, (*r* = 0.784) exhibit the strongest linear associations with overall TPACK, while the TK correlation remains comparatively weak (*r* = 0.587). These correlational patterns undermine pervasive tech-deterministic assumptions embedded within many teacher training curricula, which frame technical skill acquisition as sufficient for robust integrated instruction (Scherer et al., 2019). It should be restated that “overall TPACK” in this paper consistently denotes the seven-item integrated TPACK sub-dimension rather than an aggregate total score across all 38 scale items. Drawing on existing theoretical literature and the observed correlational pattern, PCK and TPK are tentatively labeled dual theoretical motivational-cognitive hubs in this interpretive discussion. This “hub” label represents a post-hoc theoretical framing rather than a statistical conclusion derived from correlation analysis. Read through the lens of Self-Determination Theory, PCK and TPK may theoretically operate as constructs that could concurrently afford autonomy, competence and relatedness-related experiences (Ryan and Deci, 2020). We note that our data show correlational patterns consistent with this theoretical interpretation; however, direct measurement of psychological-need satisfaction and motivational regulation would be required to empirically confirm these proposed mechanisms. PCK affords instructors agency to match pedagogical strategies to Chinese linguistic content, fulfilling autonomy needs; TPK enables adaptive digital resource selection to boost student engagement, addressing relatedness and competence demands. Trainees who receive only tool-focused training without structured curriculum design practice demonstrate weak composite TPACK scores, even with solid technical familiarity (Dong, 2023). Observational coding recorded that more than 70% of coded instructional segments consisted of PCK and TPK related observable teaching practices, while standalone technical-troubleshooting actions occurred rarely. This descriptive behavioral pattern provides supplementary observational material. The dual-hub theoretical interpretation is built upon triangulation across questionnaire correlation results, interview participant narratives and classroom observational records; classroom behavioral data on its own cannot empirically validate latent motivational-hub psychological constructs.

The identification of PCK and TPK as motivational hubs can be understood through SDT’s three basic psychological needs. PCK confidence in matching pedagogical methods to Chinese content directly satisfies the need for autonomy: when teachers possess PCK, they experience agency and volition in curriculum design, selecting approaches that align with their professional judgment rather than following prescribed formulas. TPK confidence in using technology to enhance pedagogy satisfies both competence needs (successful technology-enhanced instruction yields mastery experiences) and relatedness needs (technology enables interactive engagement with students). In contrast, TK alone addresses only a narrow, surface-level competence need (operating software) without fulfilling autonomy or relatedness, which explains its weaker association with overall TPACK. This dual-hub psychological model suggests that interventions designed to cultivate intrinsic digital teaching motivation should prioritize the integration of pedagogy with technology and content, rather than decontextualized technical skill drills.

### 5.3 Psychological mechanisms underlying demographic and experiential predictive effects

#### 5.3.1 Static demographic variables

Consistent with cross-cultural comparative analyses of language teacher motivation (Lee, 2020), gender exerted no statistically meaningful influence on any TPACK dimension (all *p* > 0.05). Caution is warranted when extrapolating this finding, however: the sample’s stark gender imbalance (21 male, 181 female participants) substantially reduces statistical power for gender-based comparisons, requiring replication with more balanced cohorts in subsequent inquiry. Age stratification analyses show trainees aged under 22 hold significantly lower PK and TCK self-efficacy than older groups (PK: *F* = 3.083, *p* = 0.028; TCK: *F* = 3.788, *p* = 0.011), a gap attributable to limited cumulative teaching practice that restricts opportunities to build competence-based confidence (Choi and An, 2021). Academic credential level showed no predictive impact on TPACK beliefs, validating the premise that hands-on instructional experience outweighs formal academic credentials for shaping technology integration self-perception (Yildiz Durak et al., 2023). Among undergraduate majors, only PCK scores differed significantly across groups: TCFL and foreign language majors reported stronger pedagogical-content confidence than peers from unrelated disciplines, a product of sustained second-language methodology coursework (Castéra et al., 2020).

#### 5.3.2 Dynamic experiential variables

The differential motivational effects of the three experiential factors can be systematically understood through the lens of SDT’s basic psychological need satisfaction.

Educational technology training is associated with significantly higher scores only in TCK (*t* = 2.613, *p* = 0.010), with no statistically significant differences in PCK, TPK, or composite TPACK. This observed pattern can be interpreted through the lens of Self-Determination Theory: tool-centric workshops may theoretically satisfy only the most rudimentary competence-related experiences, while failing to offer opportunities for autonomous curriculum design. We emphasize that basic-psychological-need satisfaction was not directly measured in the present study. The above account represents a post-hoc theoretical reading rather than empirical evidence for actual need satisfaction or motivational regulation. However, direct measurement of motivational regulation would be required to confirm this proposed mechanism.

Participants with teaching-competition experience reported higher PK-related self-perceived competence (*t* = 2.060, *p* = 0.041). Drawing on Self-Determination Theory, this pattern tentatively suggests that one-off achievement-related feedback from competitions may correspond to situational competence-related experiences. Still, competitions are episodic and externally evaluated. This theoretical framing would predict that any associated motivational gains may resemble introjected regulation and prove fragile without ongoing autonomous support. Again, since we possess no direct quantitative measures of motivational regulation or need satisfaction, this remains a theoretical conjecture rather than empirical proof.

Participants with longer cross-cultural teaching-practice experience obtained higher scores across PK, PCK, TCK and composite TPACK dimensions. From a Self-Determination Theory perspective, sustained authentic teaching practice has the theoretical potential to afford experiences related to autonomy, competence, and relatedness. We emphasize that our cross-sectional data cannot establish causal effects; group differences may stem from pre-existing participant characteristics rather than practice duration itself. The six-month tenure cut-off explored in this study should be treated as an exploratory descriptive split of our sample rather than an empirically validated critical threshold for motivational stabilization. Direct longitudinal research would be required to test whether motivational states stabilize after six-months of practice. It should be stressed that we have no direct quantitative measures of need satisfaction or motivational regulation in this dataset. The linkage between practice experience, need satisfaction and autonomous motivation constitutes a post-hoc theoretical interpretation, not an empirically verified causal conclusion.

Across all above interpretations drawing on Self-Determination Theory, it should be emphasized that SDT provides testable theoretical hypotheses to make sense of observed group differences. Since constructs of psychological-need satisfaction and motivational regulation were not directly measured within our questionnaire dataset, we are unable to empirically verify these proposed causal pathways. All theoretical inferences are clearly separated from the correlational empirical results obtained in this study.

#### 5.3.3 Hidden negative perceived contextual barriers and exploratory affective qualitative themes

No quantitative indicators of professional well-being or affective moderators were collected in this study. Beyond the quantitative TPACK findings, our semi-structured interviews yielded two exploratory qualitative themes described by participants: employment-competition pressure and cross-cultural online-teaching stress. Both themes rest on a very small evidence base. They are illustrated by two quotes drawn from eight interviews, and they were mentioned by only some of the interviewees. They are therefore reported as tentative, participant-specific accounts and carry no weight as population-level estimates. Participant narratives suggested these factors may suppress adaptive motivational functioning and create risks for professional well-being. These conclusions are derived solely from qualitative self-report accounts and cannot be generalized as population-level quantitative findings.

Employment competition pressure operates as a controlling motivational force in SDT terms. Trainees who learn digital teaching skills primarily to meet hiring benchmarks experience externally regulated motivation their behavior is driven by contingent self-worth and survival anxiety rather than intrinsic interest. This external regulation, when chronic, produces psychological depletion (Klassen and Chiu, 2010): sustained effort expended under controlling pressure exhausts self-regulatory resources, leaving teachers emotionally fatigued and diminishing their capacity for autonomous pedagogical engagement. Two of the eight interviewees described learning technologies not out of curiosity but out of fear. This is a single, small-group indication of a motivational state associated with burnout risk, and it is not sufficient to characterize the cohort.

Cross-cultural online teaching stress including time zone misalignment, unstable internet infrastructure, and intercultural communication barriers, functions as a situational need-thwarting context. Repeated instructional failures caused by these environmental constraints provide persistent negative mastery experiences, directly eroding self-efficacy beliefs (Bandura, 1997). Moreover, communication breakdowns with overseas learners impair relatedness need satisfaction, leaving trainees feeling isolated and professionally ineffective. From participants’ interview narratives, a tentative risk-related thematic pattern emerged: two of the eight interviewees described a possible self-reinforcing negative sequence: elevated teaching-related stress and self-doubt may contribute to emotional depletion, which in turn could increase subjective willingness to withdraw from TCFL-related career paths. It should be emphasized that this potential downward motivational trajectory is constructed from qualitative participant self-reports only; our cross-sectional dataset cannot empirically verify this causal spiral pathway. This pattern deserves further longitudinal empirical investigation.

### 5.4 Theoretical contributions

Three distinct theoretical advances emerge from this explanatory sequential mixed-method study. First, the analysis reframes the seven TPACK dimensions not merely as taxonomies of instructional knowledge, but as interrelated self-perceived competence constructs that we interpret as indicators of self-efficacy beliefs, theoretically linked to intrinsic and extrinsic motivational states and basic psychological need satisfaction (Dong, 2023). This reorientation expands the applied scope of Bandura’s social cognitive theory and Self-Determination Theory within TCFL teacher education research. Second, drawing on correlational patterns and prior theoretical work, this study tentatively frames PCK and TPK as discipline-specific theoretical motivational-cognitive hubs for logographic-character-based Chinese language teaching; this interpretive framing awaits further empirical validation via advanced analytical approaches, adding context-specific observations to a field dominated by English and STEM teacher samples (Zou et al., 2022). The elevated TCK profile documented here delivers novel context-specific insight into TPACK formation for character-based language teaching. This study adapts a theoretically grounded TPACK instrument for pre-service TCFL teachers and reports internal-consistency statistics. Formal empirical validation of its seven-factor structure remains a task for future research. Third, the mixed-methods design overcomes a key limit of survey-only work. It combines cognitive self-perception with affective factors that surveys cannot reach. This provides a template for future research on language teacher motivation (Creswell and Plano Clark, 2017).

### 5.5 Practical psychological intervention implications

Targeted adjustments to TCFL pre-service training curricula can be derived from the dimensional imbalance and dual-hub motivational findings. First, educational technology coursework should shift from isolated tool tutorials to integrated lesson-design modules that grant trainees autonomy to align digital resources with Chinese linguistic content, fostering intrinsic instructional motivation (Tondeur et al., 2017). Second, based on the associational patterns observed in this cross-sectional sample and previous theoretical literature, we tentatively propose that teacher-education programs could consider exploring internship arrangements of approximately six-months duration. This proposal requires further empirical testing via longitudinal research and should not be interpreted as a validated evidence-based standard derived from the current dataset (Scherer et al., 2019). In addition, drawing on interview-reported affective risk themes, we tentatively put forward two directions for future supportive-program exploration: the possibility of introducing motivational-interviewing-style counseling support and stress-reappraisal guidance for pre-service TCFL teachers. These two intervention-oriented ideas are generated from qualitative participant narratives and existing motivational theory; they have not been empirically tested within this study and require rigorous future empirical evaluation before large-scale implementation. Third, teaching competition rubrics ought to prioritize holistic technology-content-pedagogy integration rather than isolated technical demonstrations, centering PCK and TPK hub capacity development (Zou et al., 2022). Fourth, supplementary psychological support sessions addressing employment anxiety and remote teaching stress should be embedded within teacher training programs to mitigate emotional depletion and preserve sustained professional well-being (Klassen and Chiu, 2010).

Critically, the psychological mechanisms identified in this study call for targeted psychological interventions beyond curriculum redesign. First, motivational interviewing techniques can be embedded in teacher training to help pre-service teachers reflect on their motivational orientations distinguishing career-driven extrinsic motivation from genuine pedagogical interest and gradually cultivate autonomous regulation. Second, stress reappraisal training should address cross-cultural teaching anxiety by reframing performance setbacks as normative developmental challenges rather than personal failures, thereby protecting self-efficacy beliefs and reducing emotional depletion. Third, peer support networks can be structured to satisfy relatedness needs through collaborative problem-solving around cross-cultural teaching challenges, countering the isolation effects of remote instruction. These psychologically oriented interventions, integrated with existing curricular reforms, offer a more holistic pathway to sustaining pre-service teachers’ motivational functioning and long-term professional well-being.

## 6 Conclusion

This mixed-method study mapped seven-dimensional self-perceived TPACK competence among 202 pre-service TCFL teachers and examined how it relates to demographic and experiential factors. Three points summarize the contribution. All quantitative findings are cross-sectional and correlational. All qualitative findings come from eight interviews and six observed lessons and are exploratory.

First, self-perceived TPACK competence was medium-to-high but unevenly distributed. TK was lowest (M = 3.68) and TCK highest (M = 3.83), the latter associated with mandatory remote teaching rather than spontaneous interest. The composite TPACK dimension was second-lowest (M = 3.74). Because the spread across dimensions is narrow and was not formally tested, these orderings are reported descriptively.

Second, all seven dimensions correlated positively at *p* < 0.01. TPK (*r* = 0.835) and PCK (*r* = 0.784) showed the strongest associations with the integrated TPACK sub-dimension, and TK the weakest (*r* = 0.587). Classroom coding was consistent with this pattern, although coding alone cannot establish it.

Third, gender and academic level showed no significant associations. Age and undergraduate major showed limited partial effects. Teaching practice, technology training and competition experience each showed distinct associational patterns. Self-Determination Theory offers a way of reading these patterns, but need satisfaction was not measured. Interviews added two exploratory themes: employment pressure and cross-cultural online-teaching stress.

Taken together, the patterns support reading TPACK as a psychological belief system rather than only as a competency framework. Interventions that ignore autonomy, competence and relatedness are likely to produce only short-term motivation.

The study advances three things: a motivational reading of TPACK, a replicable mixed-methods template for language teacher education research, and sample-specific evidence from logographic language teaching. Practical proposals are set out in Section 5.5. They are theory-informed and untested, and they require evaluation in future intervention studies.

## 7 Limitations and future research

This study offers insights into pre-service TCFL teachers’ self-perceived TPACK competence. Several limitations restrict how far the findings generalize.

First, the sampling scope and demographic balance are constrained. All participants were recruited from normal universities in Northeast China, which cannot fully represent pre-service TCFL teacher cohorts across different regions of China with disparate teacher training systems and digital teaching resource allocations. Additionally, the sample suffers severe gender imbalance, with only 21 male participants accounting for merely 10.4% of the total sample (Lee, 2020). Such skewed gender distribution reduces the statistical power of gender difference tests, and the non-significant gender-related results cannot be treated as definitive evidence that gender exerts no influence on TPACK development. Moreover, master’s students dominate the sample (84.2%), while undergraduate and doctoral participants are far fewer, limiting the ability to compare TPACK developmental patterns across different educational stages (Yildiz Durak et al., 2023).

Second, a cross-sectional design was adopted for questionnaire data collection. All self-efficacy scores were captured at a single time point, which fails to track the dynamic developmental trajectory of TPACK beliefs over training years, internship periods and career preparation stages (Creswell and Plano Clark, 2017). This static snapshot cannot identify causal relationships between experiential factors and changes in motivational functioning; only correlational and inter-group disparities can be examined, rather than long-term causal effects.

Third, construct operationalization and measurement limitations. The TPACK scale used in this study measures self-perceived TPACK competence, participants’ subjective assessments of their own knowledge and capabilities across the seven TPACK dimensions. While we interpret these scores as indicators of technology-integration self-efficacy beliefs, this interpretation is a theoretical framing rather than a claim of psychometric equivalence. Self-efficacy, as defined by Bandura (1997), refers to individuals’ judgments of their capabilities to organize and execute courses of action required to attain designated types of performances—a more specific, context-bound construct than general perceived competence. Direct measurement of self-efficacy would require more context-specific and task-specific items that assess confidence to execute particular teaching tasks in particular situations (e.g., “How confident are you that you can use pinyin annotation software to teach tone pronunciation to beginning-level learners?”). Future research should consider developing TCFL-specific self-efficacy measures that more precisely capture Bandurian self-efficacy, or complement self-report measures with performance-based assessments of actual TPACK knowledge. Furthermore, although we followed standardized scale-adaptation workflows including expert content review, translation-back-translation and pilot testing for this TCFL-adapted 38-item instrument, the present study did not perform formal exploratory factor analysis (EFA) or confirmatory factor analysis (CFA). Consequently, the theoretically hypothesized seven-factor measurement structure has not been empirically verified in our sample. KMO and Bartlett’s Sphericity tests only assess prerequisites for factor analysis and cannot confirm factor quantity, factor structure or discriminant validity. Given our sample size N = 202, post-hoc CFA on this complex seven-factor model would likely yield unstable parameter estimates and cannot serve as rigorous structural validation. Future research should collect larger, independent samples: implement EFA first to explore latent factor patterns, followed by CFA; report factor loadings, model-fit indices, inter-factor correlations, and discriminant validity metrics (e.g., AVE, HTMT) to formally test the seven-dimensional structure of this pre-service TCFL TPACK self-perceived competence scale. Furthermore, this study’s quantitative questionnaire battery only assesses self-perceived TPACK competence alongside demographic and experiential background information. No dedicated validated quantitative instruments measuring intrinsic/extrinsic motivation, autonomy-competence-relatedness need satisfaction, emotional exhaustion or professional well-being were administered. Consequently, observed group differences in TPACK scores cannot be treated as direct empirical evidence for psychological-need satisfaction or motivational states. Interpretations invoking Self-Determination Theory serve as a theoretical interpretive lens for our quantitative patterns, rather than as hypotheses empirically tested in the present work. Evidence regarding professional well-being and affective stress-related outcomes originates exclusively from exploratory semi-structured-interview qualitative data. Future research should incorporate dedicated validated scales for motivation, psychological-need satisfaction and professional well-being to permit direct quantitative examination of these mechanisms.

Fourth, the scale relies entirely on self-reported subjective self-perception data. Although the questionnaire passed reliability tests, self-reporting inevitably carries response bias such as social desirability bias (Scherer et al., 2019). Participants may overestimate their digital teaching confidence to present a more competent self-image. Moreover, as noted above, the scale measures self-perceived competence rather than directly measuring either actual TPACK knowledge or Bandurian self-efficacy. This limitation should be considered when interpreting our findings as indicators of technology-integration self-efficacy beliefs. Furthermore, the sample size for qualitative supplementary materials is relatively small: only 8 interviewees and 6 classroom observation videos were collected, which restricts the saturation and representativeness of extracted affective moderators (Braun and Clarke, 2006).

Fifth, contextual confinement of teaching practice. All participants’ teaching experience was limited to online remote instruction shaped by pandemic restrictions, with no offline classroom practice data collected. The high TCK scores identified in this study are closely tied to this special background (Tondeur et al., 2017), and the conclusions may not fully apply to pre-service teachers who receive equal amounts of offline and online teaching training under normal educational context.

Based on the above limitations, four targeted avenues for subsequent inquiry are proposed to expand and deepen this line of research.

Expanded and balanced sampling design. Future research can adopt stratified multi-region sampling covering eastern, central and western Chinese universities to capture regional variations in digital teacher training resources. Researchers should intentionally adjust sampling quotas to recruit more male participants and balance the proportion of undergraduates, master’s and doctoral students, so as to reliably verify whether gender and academic stage exert significant effects on TPACK self-efficacy (Castéra et al., 2020). Cross-national sampling can also be considered to compare TPACK motivational differences between Chinese pre-service TCFL educators and overseas Chinese language teachers, expanding the cross-cultural applicability of the research framework.

Longitudinal mixed-method tracking research. Longitudinal panel designs with multiple waves of data collection are recommended. Researchers can track the same cohort of pre-service teachers from the first year of training to graduation and early internship, repeatedly administering the TPACK self-efficacy scale at semestral intervals (Dong, 2023). Combined with periodic follow-up interviews and periodic classroom observations, longitudinal data can clearly reveal the causal influence of training courses, teaching practice and competitions on the evolution of motivational functioning, making up for the defects of cross-sectional static data.

Diversified multi-source evaluation systems. To mitigate self-report bias, follow-up studies can build multi-dimensional evaluation systems integrating multiple data sources: combine student feedback on teachers’ digital lessons, mentor evaluation of integrated teaching design works, and objective classroom coding data with self-reported questionnaire scores (Voogt et al., 2016). Larger qualitative samples should be collected, including more interview participants and abundant offline classroom observation recordings, to dig deeper into the interactive mechanisms between employment pressure, cross-cultural teaching stress and TPACK beliefs. Structural equation modeling can be further used to quantitatively test the mediating and moderating paths of negative affective factors.

Extended research contexts and intervention research. Subsequent work can distinguish offline, hybrid and fully online teaching environments to compare differences in pre-service teachers’ TPACK dimensional structures across varied instructional scenarios. On the basis of the motivational hub mechanism uncovered in this study, intervention experiments can be carried out: design semester-long integrated pedagogy-technology training modules centered on PCK and TPK, and set blank control groups to verify whether targeted training can effectively lift pre-service teachers’ intrinsic digital teaching motivation (Zou et al., 2022). Additionally, future research can incorporate new variables such as artificial intelligence teaching tool literacy, professional identity and mental resilience, to construct a more comprehensive predictive model of pre-service TCFL teachers’ technology integration motivational functioning.

## Data Availability

The original contributions presented in the study are included in the article/supplementary material, further inquiries can be directed to the corresponding author.
