# Supplementary material for: Mapping TPACK self-efficacy beliefs in pre-service Chinese language teachers: a mixed-methods study
Source: Front Psychol. 2026 Oct 22;17:1961246. doi: 10.3389/fpsyg.2026.1961246 (PMC13736770; doi:10.3389/fpsyg.2026.1961246)
Supplement: Supplementary file 1 [file Data_Sheet_1.docx]

**Appendices**

**Appendix A** Full scale of Pre‑Service TCFL's self‑perceived TPACK competence (TPACK‑based measurement instrument)

| Title | |
| --- | --- |
| 1. TK | |
| TK1 | I am familiar with resolving common classroom technology issues (such as network connection failures, inability to play audio and video in presentation materials, etc.). |
| TK2 | I can learn technology easily. |
| TK3 | I can keep pace with the development of information technology and learn important new technologies (such as online teaching platforms). |
| TK4 | I frequently use and research technology. |
| TK5 | I am proficient in various techniques (such as video dubbing, video editing, and recording lectures). |
| TK6 | I have ample opportunities to use various technologies in my studies or work. |
| 2. CK | |
| CK1 | My knowledge in the field of Chinese as a foreign language is quite comprehensive. |
| CK2 | I am capable of delving deeply into and studying the knowledge content within the field of Chinese as a foreign language. |
| CK3 | I will reflect on my level of mastery of the knowledge in teaching Chinese as a foreign language. |
| 3. PK | |
| PK1 | I am aware of how to organize teaching and manage classrooms. |
| PK2 | I know how to evaluate the performance of Chinese learners in the classroom. |
| PK3 | I can adjust my teaching methods based on the learner's level of understanding. |
| PK4 | I can adjust teaching strategies accordingly based on the different learning styles of learners. |
| PK5 | In evaluating learners' learning progress, I am capable of employing a variety of assessment methods. |
| PK6 | I can employ diverse teaching methods during classroom instruction. |
| PK7 | I am aware of the common mistakes and difficulties students encounter when learning Chinese. |
| PK8 | I will reflect on my level of mastery in pedagogical knowledge. |
|  | 4. PCK |
| PCK1 | I am capable of selecting effective teaching methodologies when guiding students in learning Chinese. |
| PCK2 | I am capable of selecting appropriate teaching methods based on specific themes in Chinese instruction. |
| PCK3 | I believe that the integration of content and pedagogy in this discipline is conducive to students' better learning of Chinese, and I often reflect on whether the combination of the two is appropriate. |
| PCK4 | I will adjust teaching strategies to reduce students' misunderstandings of Chinese knowledge. |
| PCK5 | I am proficient in various methods of learning assessment (e.g., self-assessment, group evaluation, and examinations) and can adjust my teaching strategies based on the feedback from these assessments.   5. TCK |
| TCK1 | I have a good understanding of the information technology resources that can be used for Chinese language teaching. |
| TCK2 | I am capable of selecting appropriate technologies, such as animation effects and PowerPoint, to represent specific knowledge points, facilitating better understanding for learners. |
| TCK3 | I will appropriately modify existing technical resource products (such as audio-visual materials, courseware, etc.) according to the needs of the teaching content. |
| TCK4 | I am capable of teaching Chinese online. |
|  | 6. TPK |
| TPK1 | I can select different techniques (such as creating demonstration animations) to enhance the effectiveness of my teaching methods in the classroom. |
| TPK2 | I know how to select appropriate technology to engage learners and enhance their classroom learning experience. |
| TPK3 | The teacher education courses I have taken have helped me delve deeper into how information technology impacts the pedagogy I employ. |
| TPK4 | I am capable of critically evaluating the appropriateness of technology use in Chinese language classroom instruction. |
| TPK5 | I am capable of processing and adapting the techniques I learn to suit a variety of different teaching scenarios.   7. TPACK |
| TPACK1 | I am capable of appropriately integrating information technology, pedagogy, and knowledge of teaching Chinese as a foreign language in my teaching. |
| TPACK2 | I can select appropriate technologies based on how learners learn, what is taught, and how it is taught in the classroom. |
| TPACK3 | I can use strategies in the classroom, integrating technology, teaching methods, and subject content. |
| TPACK4 | I am capable of enhancing classroom teaching effectiveness by employing diverse techniques tailored to different teaching content. |
| TPACK5 | I can take a leadership role in assisting others with the coordination and utilization of technology, instructional methods, and subject matter content. |
| TPACK6 | I can utilize technology to create authentic contexts related to the content in classroom teaching, which can engage students' interest and stimulate their learning motivation. |
| TPACK7 | I will reflect on the integration of content, pedagogy, and technology in my discipline, and whether their application in the classroom is appropriate. |

Note: This is an adapted instrument for pre‑service TCFL; its theoretical seven‑dimensional factor structure awaits empirical verification in future factor‑analytic research.

**Appendix B** Basic demographic information of 8 interviewees

| Basic Information | A | B | C | D | E | F | | G | H |
| --- | --- | --- | --- | --- | --- | --- | --- | --- | --- |
| Gender | Female | Female | Female | Male | Female | Female | | Female | Male |
| Educational technology courses/training | Yes | Yes | Yes | Yes | Yes | Yes | | Yes | Yes |
| Undergraduate major | Korean | TCFL | TCFL | TCFL | Chinese Language and Literature | English | Chinese Language and Literature | | TCFL |
| Teaching competition experience | Yes | Yes | Yes | Yes | Yes | Yes | | Yes | Yes |
| Teaching experience | Yes | Yes | Yes | Yes | Yes | Yes | | Yes | Yes |
| Teaching identity | Training institution CFL teacher | CFL teacher volunteer | School project CFL teacher | School project CFL teacher | School project CFL teacher | School project CFL teacher | | Training institution CFL teacher | CFL teacher volunteer |

(Note: TCFL = Teaching Chinese as a Foreign Language)

Participants A, B, C and D contributed to both qualitative components: each completed a semi-structured interview and also provided a classroom-teaching video (see Appendix C). Participants E, F, G and H completed interviews only. The remaining two classroom videos were contributed by participants who did not take part in the interviews. This mapping is provided so that readers can judge how far the interview and observation strands can be triangulated for any given participant.

**Appendix C** Full classroom motivational behavior coding form

| Code | Dimension | Description | Example |
| --- | --- | --- | --- |
| TK | Technological Knowledge | Knowledge of traditional and modern information technology. Coded when teachers use or operate technology without integrating it with content or pedagogy. | "Please look at the pictures on the screen"; teacher checking students' homework via software; adjusting audio settings. |
| CK | Content Knowledge | Knowledge of subject content, including concepts and theories. Coded when teachers present subject content alone. | "The reduplication forms of verbs and adjectives are different"; teacher writing "attribute," "complement," "Spring Festival" on the board. |
| PK | Pedagogical Knowledge | Knowledge of teaching processes, methods, and practices. Coded when teachers use specific teaching methods or strategies. | "Today we will study Lesson 5..."; "Thank you," "Please sit down," "Excellent"; teacher asking a student or group to answer a question. |
| PCK | Pedagogical Content Knowledge | Knowledge of using teaching methods to facilitate student learning of specific content. Coded when teachers link pedagogy with subject content. | "Please open your textbooks to pages 6-10 and discuss in groups"; "Please make a sentence based on the example on the board"; teacher eliciting students' holiday activities to introduce a new grammar point. |
| TCK | Technological Content Knowledge | Knowledge of presenting subject content through technology. Coded when teachers use technology to present content. | "This is the structure of the 'ba' construction"; "Please look at the knowledge points on the left side of the screen and summarize today's lesson." |
| TPK | Technological Pedagogical Knowledge | Knowledge of using technology to enhance teaching effectiveness. Coded when teachers link technology with pedagogical strategies. | "To help you master this, I have provided several websites"; teacher using random name selection software to call on students. |
| TPACK | Technological Pedagogical Content Knowledge | Knowledge of integrating technology, pedagogy, and content. Coded when teachers integrate all three core elements. | Teacher simultaneously explaining content and operating the computer, using a video to demonstrate the process of making dumplings while teaching the construction. |
| N | Null | Teacher behaviours unrelated to the seven TPACK dimensions. | Casual greetings with students that do not involve teaching content. |

**Appendix C** Detailed Information of Six Observed Teaching Videos

| **Teacher Code** | **Teaching Content** | **Duration** |
| --- | --- | --- |
| A | "There is no place more beautiful than that" | 40'16" |
| B | "HSK Level 6 Writing 1" | 43'34" |
| C | "Places with mountains and water" | 36'49" |
| D | "Little painter in the snow" | 35'01" |
| E | "Do not do to others what you do not want others to do to you" | 35'10" |
| F | "Picturesque Sun Moon Lake (Part 1)" | 34'52" |

**Supplementary Materials**

Table S1 Participant demographic characteristics (N = 202)

| **Variable** | **Category** | **n** | **Percentage (%)** |
| --- | --- | --- | --- |
| Gender | Male | 21 | 10.4 |
|  | Female | 181 | 89.6 |
| Age | ＜22 years | 17 | 8.4 |
|  | 22‑25 years | 157 | 77.7 |
|  | 26‑28 years | 25 | 12.4 |
|  | ＞28 years | 3 | 1.5 |
| Educational level | Undergraduate | 30 | 14.9 |
|  | Master’s student | 170 | 84.2 |
|  | Doctoral student | 2 | 1.0 |
| Undergraduate major | International Chinese education | 141 | 69.8 |
|  | Chinese language and literature | 21 | 10.4 |
|  | Foreign language disciplines | 29 | 14.4 |
|  | Other majors | 11 | 5.4 |
| Received educational‑technology training | Yes | 128 | 63.4 |
|  | No | 74 | 36.6 |
| Participated in Chinese‑teaching skill competitions | Yes | 108 | 53.5 |
|  | No | 94 | 46.5 |
| Practical Chinese‑teaching experience | Yes | 149 | 73.8 |
|  | No | 53 | 26.2 |

Table S2 Descriptive statistics for seven TPACK dimensions

| **Dimension** | **N** | **Min** | **Max** | **M** | **SD** | **Median** |
| --- | --- | --- | --- | --- | --- | --- |
| TK | 202 | 2.33 | 5.00 | 3.68 | 0.58 | 3.67 |
| CK | 202 | 1.00 | 5.00 | 3.81 | 0.65 | 4.00 |
| PK | 202 | 2.00 | 5.00 | 3.80 | 0.59 | 3.88 |
| PCK | 202 | 2.00 | 5.00 | 3.80 | 0.55 | 3.90 |
| TCK | 202 | 2.00 | 5.00 | 3.83 | 0.63 | 4.00 |
| TPK | 202 | 2.00 | 5.00 | 3.79 | 0.63 | 3.80 |
| TPACK | 202 | 2.00 | 5.00 | 3.74 | 0.58 | 3.86 |

Table S3 Pearson correlations between TPACK dimensions (all p < 0.01)

| **Pairwise comparison** | **Correlation coefficient r** |
| --- | --- |
| TPK — TPACK | 0.835 |
| PCK — TPACK | 0.784 |
| PK — TPACK | 0.781 |
| TCK — TPACK | 0.769 |
| CK — TPACK | 0.639 |
| TK — TPACK | 0.587 |

Table S4 Summary of group‑difference analyses

| **Grouping variable** | **Significant TPACK dimension** | **Statistic** | **df** | **p** | | **Effect‑size** | **95 % CI** | **Observed pattern** |
| --- | --- | --- | --- | --- | --- | --- | --- | --- |
| Gender | None | t | 199 | >0.05 | d = 0.11 | | [-0.13, 0.21] | No significant association |
| Age | PK, TCK | F | 2, 199 | PK = 0.028；TCK = 0.011 | η² (PK)=0.031；η² (TCK)=0.038 | | PK [0.001, 0.080]; TCK [0.003, 0.089] | Participants < 22 years scored lower |
| Undergraduate major | PCK | F | 3, 198 | 0.046 | η² = 0.027 | | [0.000, 0.073] | Differences across major categories |
| Educational‑technology training | TCK | t | 200 | 0.010 | d = 0.37 | | [0.09, 0.66] | Trained participants scored higher |
| Teaching‑skill competition experience | PK | t | 200 | 0.041 | d = 0.28 | | [0.01, 0.55] | Competition‑exposed participants scored higher |
| Chinese‑teaching practical experience | PK, PCK, TCK, TPACK | t | 200 | PK = 0.002; PCK = 0.004; TCK = 0.006; TPACK = 0.048 | d(PK)=0.44；d(PCK)=0.40；d(TCK)=0.38；d(TPACK)=0.28 | | PK [0.16, 0.72]; PCK [0.12, 0.68]; TCK [0.10, 0.66]; TPACK [0.01, 0.55] | Participants with experience scored higher |
| Teaching tenure (only participants with teaching experience, n = 149) | TK, CK, PK, PCK, TCK, TPK, TPACK | H | 3 | < 0.05 | ε² range 0.07‑0.13 | | — | Longer tenure associated with higher scores |
| Teaching identity | None | H | 3 | >0.05 | ε² < 0.03 | | — | No significant association |

Notes. Bonferroni correction was applied for multiple testing. Cohen’s d for t‑tests; η² for ANOVA; ε² for Kruskal‑Wallis H‑test. Small‑subgroup analyses (n ≤ 20) are interpreted as exploratory patterns.
